# A Genomic Profile of Local Immunity in the Melanoma Microenvironment Following Treatment with α Particle-Emitting Ultrasmall Silica Nanoparticles

**DOI:** 10.1089/cbr.2019.3150

**Published:** 2020-08-13

**Authors:** Aleksandra M. Urbanska, Raya Khanin, Simone Alidori, Sam Wong, Barbara P. Mello, Bryan Aristega Almeida, Feng Chen, Kai Ma, Melik Z. Turker, Tatyana Korontsvit, David A. Scheinberg, Pat B. Zanzonico, Ulrich Wiesner, Michelle S. Bradbury, Thomas P. Quinn, Michael R. McDevitt

**Affiliations:** ^1^ Department of Radiology, Memorial Sloan Kettering Cancer Center, New York, New York, USA.; ^2^ Bioinformatics Core Computational Biology Program, Memorial Sloan Kettering Cancer Center, New York, New York, USA.; ^3^ Department of Chemistry, Hunter College, New York, New York, USA.; ^4^ Molecular Pharmacology Program, Memorial Sloan Kettering Cancer Center, New York, New York, USA.; ^5^ Department of Materials Science & Engineering, Cornell University, Ithaca, New York, USA.; ^6^ Department of Pharmacology, Weill Cornell Medicine College, New York, New York, USA.; ^7^ Department of Medical Physics, Memorial Sloan Kettering Cancer Center, New York, New York, USA.; ^8^ Department of Radiology, Weill Cornell Medical College, New York, New York, USA.; ^9^ Department of Biochemistry, University of Missouri, Columbia, Missouri, USA.; ^10^ Harry S. Truman Veterans' Hospital, Columbia, Missouri, USA.

**Keywords:** ultrasmall silica nanoparticles, C′ dot, immunotherapy, α particle, Actinium-225, melanoma, macrophage, T cell, natural killer cell, pseudopathogen, tumor microenvironment

## Abstract

An α particle-emitting nanodrug that is a potent and specific antitumor agent and also prompts significant remodeling of local immunity in the tumor microenvironment (TME) has been developed and may impact the treatment of melanoma. Biocompatible ultrasmall fluorescent core–shell silica nanoparticles (C′ dots, diameter ∼6.0 nm) have been engineered to target the melanocortin-1 receptor expressed on melanoma through α melanocyte-stimulating hormone peptides attached to the C′ dot surface. Actinium-225 is also bound to the nanoparticle to deliver a densely ionizing dose of high-energy α particles to cancer. Nanodrug pharmacokinetic properties are optimal for targeted radionuclide therapy as they exhibit rapid blood clearance, tumor-specific accumulation, minimal off-target localization, and renal elimination. Potent and specific tumor control, arising from the α particles, was observed in a syngeneic animal model of melanoma. Surprisingly, the C′ dot component of this drug initiates a favorable pseudopathogenic response in the TME generating distinct changes in the fractions of naive and activated CD8 T cells, Th1 and regulatory T cells, immature dendritic cells, monocytes, MΦ and M1 macrophages, and activated natural killer cells. Concomitant upregulation of the inflammatory cytokine genome and adaptive immune pathways each describes a macrophage-initiated pseudoresponse to a viral-shaped pathogen. This study suggests that therapeutic α-particle irradiation of melanoma using ultrasmall functionalized core–shell silica nanoparticles potently kills tumor cells, and at the same time initiates a distinct immune response in the TME.

## Introduction

First-generation ultrasmall fluorescent core–shell silica nanoparticles (Cornell dots [C dots]) and their next-generation analogs (Cornell prime dots [C′ dots]) have hydrodynamic diameters below 10 nm and silica core-encapsulated near-infrared-emitting dyes.^1–11^ These innovative nanoscale materials have been engineered to target cancer and deliver therapeutic payloads *in vivo* as well as image tissue distribution and clearance.^1–11^ C′ dots that do not target disease are readily eliminated through renal filtration due to their ultrasmall particle dimensions.^9,10^ These novel particulate materials have a performance advantage that has been demonstrated in animal models and consequently accelerated their translation into humans. The Phase 1 first-in-human clinical trial using the FDA-approved Investigational New Drug hybrid core–shell silica particle, [^124^I]cRGDY-PEG-C dot, is an iodinated drug that targets tumor, eliminates in urine, and is safe and biocompatible in man.^5^

Particle design has been advanced beyond the first-generation nanomaterials by appending of α melanocyte-stimulating hormone (αMSH) analog peptide sequences designed to target the melanocortin-1 receptor (MC1R) expressed on melanoma.^8,12^ Additional synthetic modifications include covalent attachment of chelating agents preloaded with the α particle-emitting actinium-225 (^225^Ac) radionuclide to deliver a cytotoxic dose of radiation to the tumor.^13,14^ MC1R targeting of metastatic melanoma in humans using a small-molecule αMSH peptide has been reported and demonstrates the clinical relevance of MC1R.^15^

Actinium-225 (*t*_1/2_ = 10 d) deposits a high dose of energy (5–8 MeV) over a short range (50–80 μm), producing specific and potent cytotoxicity^16^ and has been used clinically to treat leukemia with the antibody lintuzumab.^17–19^ High linear energy transfer α particles are lethal to cancer cells as a consequence of ineffective double-strand DNA repair.^20,21^ Moreover, in internalizing systems, such as αMSH-PEG-Cy5-C′ dot and Lintuzumab, each ^225^Ac decay produces several daughters generating three additional α particles able to contribute to cytotoxicity.^13^

Malignant melanoma is diagnosed in ∼90,000 individuals in the United States per year and is the most lethal form of skin cancer.^22^ The incidence of disease has increased rapidly over the past 50 years. Melanoma is an aggressive disease and metastatic stage-IV melanoma is difficult to treat despite recent advances in immunotherapies.^23–26^ Median survival ranges from 8 to 12 months with standard-of-care treatment, including immunotherapeutic drugs such as ipilimumab and nivolumab.^23–26^ Novel nanomolecular drug agents constructed from silica permit MC1R targeting and now enable selective delivery of the α-particle emitters actinium-225, yielding a potent new treatment option for metastatic melanoma. Close inspection of the tumor microenvironment (TME) following irradiation of disease now reveals that the immune cell composition, cytokine mRNA, and inflammatory pathways undergoes dynamic changes arising from the C′ dot platform.

Radiotherapy can upregulate cytokine signaling and inflammatory cascades^27^ and nanomaterials have been recognized as contributing factors in modifying the immune milieu.^28–31^ Novel nanoplatforms require further investigation to harness their full potential. Herein, we now describe the pharmacology of the radiotherapeutic α particle-emitting [^225^Ac]αMSH-PEG-Cy5-C′ dot drug and describe the unexpected contribution of the αMSH-PEG-Cy5-C′ dot nanoparticle platform in an immunocompetent, syngeneic mouse model of melanoma. This study describes downstream effects on TME immune cell populations, cytokine expression, and inflammatory pathways arising from an α particle-emitting ultrasmall fluorescent core–shell silica nanoparticle and explores the unique combination of α particles and targeted C′ dot-based adjuvant immunotherapeutic approaches to eliminate melanoma.

## Materials and Methods

### Chemical synthesis and physical characterization of ultrasmall αMSH-functionalized C′ dot precursors

The synthesis, characterization, and biology of αMSH-PEG-Cy5-C′ dots and nontargeting control NH_2_-PEG-Cy5-C′ dots have been described.^8^ The precursor αMSH-PEG-Cy5-C′ dot particle was synthesized as follows: Heterobifunctional *N*-hydroxysuccinimide ester polyethylene glycol maleimide (860 g/mol, 12 ethylene glycol units per molecule; Quanta Biodesign) was reacted at ambient temperature with (3-aminopropyl)triethoxysilane (Sigma-Aldrich) under nitrogen to form Mal-PEG-silane. The αMSH peptide was subsequently added to the Mal-PEG-silane at ambient temperature under nitrogen to produce αMSH-PEG-silane. The Cy5-silane component was prepared by conjugating maleimido-functionalized Cy5 dyes (GE Healthcare Life Sciences) with (3-mercaptopropyl)trimethoxysilane (Sigma-Aldrich) at ambient temperature under nitrogen. Tetramethyl orthosilicate (Sigma-Aldrich) and Cy5-silane were then mixed in an aqueous ammonium hydroxide solution (pH adjusted to 8.5) at ambient temperature with stirring. αMSH-PEG-silane and monofunctional PEG-silane (∼500 g/mol, 6–9 ethylene glycol units; Gelest) were added into the reaction at ambient temperature with stirring overnight. The resulting αMSH-PEG-Cy5-C′ dots were dialyzed against deionized water, purified using gel permeation chromatography (GPC), and filtered by sterile syringe filters. The final product was characterized and stored at 4°C. GPC purification and characterization of the particles was conducted using a Biologic LP system (Bio-Rad) equipped with a 275-nm ultraviolet detector and a chromatography column packed with Superdex 200 resin (GE Healthcare Life Sciences). Fluorescence correlation spectroscopy (FCS) measurements were conducted using a homebuilt FCS instrument with a 633-nm solid-state laser as the excitation source.^8^

### Internalization of αMSH-PEG-Cy5-C′ dots by macrophages and B16-F10 tumor cells measured using fluorescence-activated cell sorting

In fluorescence-activated cell sorting (FACS) Study 1, C57BL/6J mice (♀; 8–12 weeks old; Jackson Laboratory, Bar Harbor, ME) were implanted with 5 × 10^5^ B16-F10 cells through subcutaneous (SC) injection and separated into two groups. Each animal received an intravenous (IV) injection of 50 pmol of αMSH-PEG-Cy5-C′ dots formulated in 1% human serum albumin (HSA; Swiss Red Cross)/0.9% NaCl (Abbott Laboratories) (1% HSA) or only the 1% HSA vehicle (0 pmol C′ dots) through retro-orbital sinus injection under anesthesia 8 d after B16-F10 implantation. The mice were euthanized 4–5 d postadministration of C′ dots and tumor harvested and dissociated into single-cell suspensions using the Tumor Cell Isolation Kit (catalog #130-096-730; Miltenyi Biotec) for 45 min at 37°C with shaking. The single-cell suspensions were individually passed through a 70 μm strainer to isolate single cells, pelleted, and resuspended in RPMI media. The extracted cells were analyzed by FACS (LSR Fortessa; BD Biosciences) to measure C′ dot internalization by tumor macrophages and melanoma cells. In FACS Study 2, naive, immunocompetent C57BL/6J mice (♀; 8–12 weeks old; Jackson Laboratory) each received an intraperitoneal (IP) injection of 50 pmol of αMSH-PEG-Cy5-C′ dots in 1% HSA or vehicle alone. Macrophages were isolated from the IP cavity at 48 h and analyzed by FACS to measure C′ dot internalization. In both of these studies *in vivo*, the harvested cells were blocked with mouse FcR Blocking Reagent (Miltenyi Biotec); macrophages were stained with PE/Cy7 anti-mouse F4/80 (#123113; BioLegend); B16-F10 tumor cells were stained with PE/Cy7 anti-mouse Podoplanin (PDPN, #127411; BioLegend); C′ dots exhibit Cy5 fluorescence; and all cells were stained with 1 g/L 4′,6-diamidino-2-phenylindole (DAPI; Sigma-Aldrich). Compensation controls were performed using single-color staining of cells or UltraComp eBeads compensation beads (Thermo Fisher Scientific). In FACS Study 3, wild-type THP-1 cells (THP-1^wt^), phorbol 12-myristate 13-acetate (PMA; Sigma-Aldrich) differentiated THP-1 cells (THP-1^PMA^), and B16-F10 cells were treated with 25 pmol of αMSH-PEG-Cy5-C′ dots in phosphate-buffered saline (PBS) or only the PBS vehicle (0 pmol C′ dots) and analyzed by FACS to measure C′ dot internalization at 20, 48, 72, and 96 h. All data were acquired with the LSR Fortessa using FACSDIVA software (version 8.0.1; BD Biosciences) and analyzed using FlowJo software (version 10.5.3 for Mac; Tree Star, Inc.), excluding debris and doublets using light scatter measurements and dead cells by DAPI staining.

### ^225^Actinium radiochemical labeling of C′ dots and quality control

A two-step radiochemical labeling methodology was employed to prepare ^225^Ac-labeled αMSH-PEG-Cy5-C′ dots.^13,14^ Briefly, 37 MBq (1 mCi) of acidic ^225^Ac nitrate (U.S. Department of Energy [ORNL, TN]) dissolved in 0.2 M HCL (Fisher Scientific) was added to a solution of 0.5–1.0 mg of S-2-(4-isothiocyanatobenzyl)-1,4,7,10-tetraazacyclododecane tetraacetic acid (DOTA-Bz-SCN; Macrocyclics, Inc.) in 0.10 mL metal-free water. The pH was adjusted with the addition of 0.1 mL of 2 M tetramethylammonium acetate (Sigma-Aldrich) and 0.02 mL of 150 g/L l-ascorbic acid (Sigma-Aldrich) to yield a pH 5.5 reaction mixture. The reaction was heated at 55°C–60°C for 30 min. An aqueous solution of the αMSH-PEG-Cy5-C′ dots (3 nmol in 0.225 mL water) was added to the [^225^Ac]DOTA-Bz-SCN reaction mixture and the pH adjusted to 9.5 with the addition of 0.15 mL of 1 M carbonate/bicarbonate buffer solution (Fisher Scientific). The reaction was held at 37°C for 30–60 min and subsequently quenched with 0.020 mL of 50 mM diethylenetriaminepentaacetic acid (Sigma-Aldrich). The reaction mixture was purified by size exclusion chromatography (SEC) using a P6 resin (Bio-Rad) as the stationary phase and 1% HSA as mobile phase. The radiochemical purity of the final radiolabeled product, [^225^Ac]αMSH-PEG-Cy5-C′ dot, was determined by instant thin-layer chromatography using silica gel. ^225^Ac activity was assayed in a Squibb CRC-15R Radioisotope Calibrator (E.R. Squibb and Sons, Inc.) set at 775, and the displayed activity multiplied by 5 at secular equilibrium. ^225^Ac-radiolabeled NH_2_-PEG-Cy5-C′ dots used in control experiments were prepared through attaching [^225^Ac]DOTA-Bz-SCN to the primary amine groups on the surface of NH_2_-PEG-Cy5-C′ dots using an approach referred to as post-PEGylation surface modification by insertion^32^ and the resulting [^225^Ac]NH_2_-PEG-Cy5-C′ dots were quality controlled in the same way as the targeted dots.

### Pharmacokinetic studies

Tissue biodistribution and clearance studies were performed using an immunocompetent C57BL/6J mouse model (♀; 6–8 weeks old; Jackson Laboratory). The tissue distribution, blood compartment clearance, and renal excretion of [^225^Ac]αMSH-PEG-Cy5-C′ dot were measured in both (a) healthy and (b) B16-F10 tumor-bearing animals. Tumors were initiated with a SC injection of 10^5^ B16-F10 cells. All experiments were done in accordance with the guidelines of the National Institutes of Health on the care and use of laboratory animals and all protocols were approved by the Memorial Sloan Kettering Institutional Animal Care and Use Committee. Healthy naive mice received an IV injection of 11.1 kBq (300 nCi) of [^225^Ac]αMSH-PEG-Cy5-C′ dots through retro-orbital sinus injection under anesthesia (*n* = 3 per time point) and were euthanized and tissues, blood, and urine harvested at 1, 24, 48, 72, and 144 h postinjection. Mice with SC melanoma received an IV injection of 11.1 kBq (300 nCi) of [^225^Ac]αMSH-PEG-Cy5-C′ dot through retro-orbital sinus injection under anesthesia (*n* = 5 per group) and were euthanized with tissues, blood, and urine harvested at 1, 24, 96, and 120 h postinjection. The tissue samples were weighed and the ^225^Ac activity measured at secular equilibrium using a γ-counter (COBRA II; Packard Instrument Company, Meriden, CT). The 370–520 keV energy window was used to quantitate the activity per tissue. Samples of each injectate formulation were used as decay correction standards. Data were expressed as %ID/g. Aliquots of the injected drug (0.020 mL) were used as decay correction standards. The percentage of the injected dose of [^225^Ac]αMSH-PEG-Cy5-C′ dot per gram of tissue weight (%ID/g) was calculated for each animal, decay-corrected to the time of injection, and the mean %ID/g was determined at each time point.

### Absorbed dose estimates

The absorbed doses to tissues from [^225^Ac]αMSH-PEG-Cy5-C′ dot were estimated from %ID/g values derived from the foregoing biodistribution data. For each tissue, the values were plotted versus the time postinjection and fit to an exponential function. The resulting time–activity functions were then analytically integrated, incorporating the effect of the radioactive decay, to obtain the tissue residence times (MBq-s/MBq administered) of ^225^Ac. For each tissue, the absorbed dose (in cGy/MBq of ^225^Ac administered) in mice was then calculated by multiplying the tissue residence time concentration (MBq-s/kg) by the ^225^Ac equilibrium dose constant for nonpenetrating radiations (α particles), 9.39 × 10^−11^ cGy-kg/MBq-s, assuming complete local absorption of the α particles and ignoring the very small β-particle and γ-ray dose contribution. The ^225^Ac tissue residence times in the 70-kg Reference Man anatomic model were obtained by inverse scaling based on the body masses of the Reference Man and a 25g mouse and using the Reference-Man tissue masses. Reference-Man tissue absorbed doses were then calculated using the *OLINDA/EXM* internal-radionuclide dosimetry computer program.

### Determination of the maximum tolerated ^225^Ac dose

Naive, immunocompetent C57BL/6J mice (♀; 6–8 weeks old; Jackson Laboratory) were randomized to four separate groups (*n* = 5). Animals in Groups I, II, III, and IV each received an IV injection of 0, 23.1, 46.3, or 92.5 kBq of [^225^Ac]αMSH-PEG-Cy5-C′ dot, respectively, through retro-orbital sinus injection under anesthesia. The animals were monitored regularly to assess overall health and were weighed weekly for 4 weeks. Toxicity was scored when body weight loss was ≥10% compared with baseline or there was severe lethargy or death. Survival data were analyzed by the Kaplan–Meier method using Prism software.

### Pharmacodynamic studies

Radiotherapeutic α particle effects on tumor growth and animal survival were assessed using immunocompetent C57BL/6J mice (♀ and ♂; 6–8 weeks old; Jackson Laboratory). Briefly, each animal received SC injections of 10^5^ B16-F10 cells and 8 d later were randomly sorted into 3 groups of 10 animals (5♀ and 5♂ per group). Mice in each group received an IV injection of 11.1 kBq of [^225^Ac]αMSH-PEG-Cy5-C′ dot (Group I), 11.1 kBq of [^225^Ac]NH_2_-PEG-Cy5-C′ dot (Group II), or the 1% HSA injection vehicle (Group III) through retro-orbital sinus injection under anesthesia. The specific activity of the injected C′ dots is 227,484 ± 57,583 GBq/mol (*n* = 6) and 55 ± 12 pmol was injected. Tumor dimensions were measured using calipers and the volume calculated using the formula: volume = 1/2(length × width^2^). Mice were sacrificed when tumor was >2000 mm^3^ or if they exhibited lethargy. Survival was plotted using the Kaplan–Meier method and tumor samples from representative animals were harvested for histopathology.

### Immune cells populating the α-irradiated TME

Immunofluorescence (IF) staining of tumor tissue harvested from the Group I [^225^Ac]αMSH-PEG-Cy5-C′ dot-treated mice and Group III vehicle-treated mice (see Pharmacodynamic Studies section above) was performed to image the kinetics of immune cell in the TME post-treatment. Representative animals were euthanized at 1, 24, 96, and 120 h post-treatment and harvested tumor was fixed in 4% paraformaldehyde/PBS for 24 h. Fixed tissue was paraffin embedded and cut into 5-μm sections and mounted for imaging. IF staining was performed at the MSKCC Molecular Cytology Core Facility using a Discovery XT processor (Ventana Medical Systems). Stains used include anti-CD3 (0.5 μg/mL, #A0452; eBioscience) and anti-IBA1 (0.4 μg/mL, #091-19741; Vector). Tumor tissue sections were scanned using a Mirax digital slide scanner (Carl Zeiss Microimaging) with the × 20 lens and analyzed with Pannoramic Viewer software and quantification of areas stained positive for T cells and macrophages was performed using Fiji imaging software.^33,34^

### Transcriptome sequencing of CD45-positive immune cells isolated from treated tumor

Briefly, C57BL/6J mice (11♂ and 11♀) received SC injections of 10^5^ B16-F10 cells and 8 d later were randomly placed into three groups. Transcriptome sequencing Group I received only an IV injection of 1% HSA vehicle (*n* = 6; 3♀ and 3♂) through retro-orbital sinus injection under anesthesia; Group II received an IV injection of 11.1 kBq of [^225^Ac]αMSH-PEG-Cy5-C′ dot (*n* = 10; 5♀ and 5♂); and Group III received an IV injection of unlabeled αMSH-PEG-Cy5-C′ dot (*n* = 6; 3♀ and 3♂). Based on the immune cell imaging analyses (see Immune cells populating the a-irradiated TME section), all mice were euthanized 96 h post-treatment and tumor harvested. The tumor was dissociated into single-cell suspensions using the Tumor Cell Isolation Kit (catalog #130-096-730; Miltenyi Biotec) for 45 min at 37°C with shaking. The single-cell suspensions were individually passed through a 70 μm strainer to isolate single cells, pelleted, and resuspended in RPMI media. CD45 Microbeads (catalog #130-052-301; Miltenyi Biotec) were added to separate CD45-positive (CD45^+^) cells from the suspension. The CD45^+^ cells isolated from tumor were counted and stored at −80°C in TRIzol. RNA was extracted from cells with chloroform, isopropanol, and linear acrylamide added, and the RNA was precipitated with 75% ethanol. The sample was resuspended in RNase-free water and quality controlled using an Agilent BioAnalyzer. Transcriptome sequencing used 500 ng of total RNA from each tumor, which underwent polyA selection and TruSeq library preparation according to instructions provided by Illumina (TruSeq Stranded mRNA LT Kit, catalog #RS-122-2102), with eight cycles of PCR. Samples were barcoded and run on a HiSeq 4000 or HiSeq 2500 in rapid mode in a 50 bp/50 bp paired-end run, using the HiSeq 3000/4000 SBS Kit or HiSeq Rapid SBS Kit v2 (Illumina). An average of 46 million paired reads was generated per sample. Ribosomal reads were not detectable, and the percent of mRNA bases averaged 74%.

### Bioinformatics pipeline

Output data (FASTQ files) were mapped to the mouse genome (Genome: UCSC MM10) using the rnaStar aligner^35^ that maps reads genomically and resolves reads across splice junctions. A 2-pass mapping method was employed in which reads are mapped twice.^36^ The first mapping pass uses a list of known annotated junctions from Ensemble. Novel junctions found in the first pass are then added to the known junctions and a second mapping pass is done (n.b., on the second pass the RemoveNoncanoncial flag is used). After mapping, the output SAM files were postprocessed using the PICARD tools to add read groups, AddOrReplaceReadGroups, which in addition sorts the files and converts them to the compressed BAM format. The expression count matrix was computed from the mapped reads using HTSeq (www-huber.embl.de/users/anders/HTSeq) and mouse gene model database (GTF:Mus_musculus.GRCm38.80). The raw count matrix generated using HTSeq was then processed using the R/Bioconductor package DESeq (www-huber.embl.de/users/anders/DESeq), which is used to both normalize the full dataset and analyze differential expression between sample groups. Heatmaps were generated using the heatmap.2 function from the gplots R package. For (a) the top 100 differentially expressed gene and (b) top 71 differentially expressed cytokine Heatmaps, a cutoff of FC = 2 and FDR = 0.05 were used and the data were plotted as the mean centered normalized log2 expression.

### Computational biology

Transcriptome data obtained from the CD45^+^ cells isolated from tumor were used to infer mouse immune signatures, cytokine expression, and pathways. Three phenotype classes were considered for this analysis: (a) a vehicle-treated control group (*n* = 6), (b) the [^225^Ac]αMSH-PEG-Cy5-C′ dot-treated group (*n* = 9), and (c) an unlabeled αMSH-PEG-Cy5-C′ dot-treated control group (*n* = 6). Briefly, the CIBERSORT deconvolution method^37^ and ImmuneCC signatures^38^ were used to calculate the relative immune cell fractions. Specifically, CIBERSORT was run on the normalized counts matrix using mouse signature genes derived from the ImmuneCC signature. Some genes from the immune signature matrix were not present in the count matrix (i.e., they had zero counts across all samples in these experiments) and were excluded from the analysis. Pathway enrichment analysis was performed by DAVID functional annotation tool https://david.ncifcrf.gov

### Statistical analyses

Graphs were constructed using Prism (GraphPad Software, Inc.) and Kaplan–Meier analysis applied for survival curve analysis. Statistical comparisons between the experimental groups were performed by Student's *t*-test (unpaired, two-tailed), or log-rank/Mantel–Cox test depending on the analysis. Multiple *t*-test analysis of the immune cell fractions used the method of Benjamini, Krieger, and Yekutieli to examine *p*-value distributions and estimate the fraction of true null hypotheses using a false discovery rate of 1%.

## Results

**FIG. 1.**
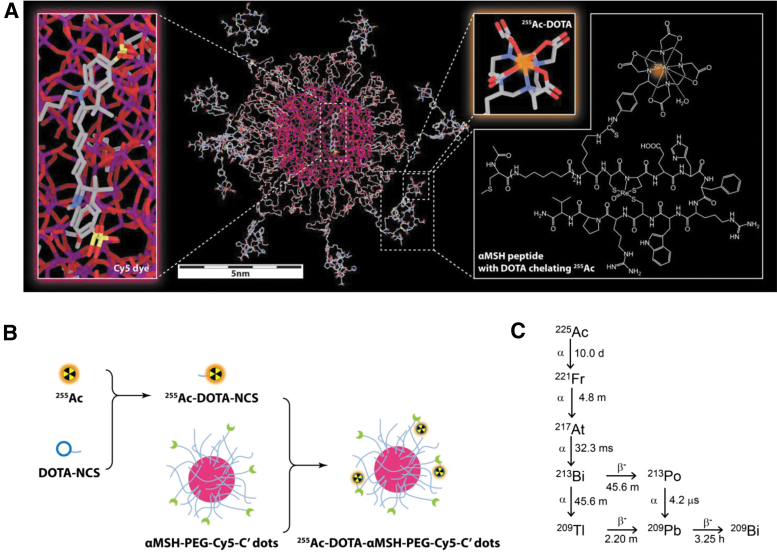
Molecular structure of [^255^Ac]αMSH-PEG-Cy5-C′ dots, radiochemical process, and ^225^Ac decay scheme. **(A)** An illustration of the particle composition with silicon, oxygen, carbon, nitrogen, sulfur, and actinium atoms color coded as *purple*, *red*, *gray*, *blue*, *yellow,* and *orange*, respectively. Hydrogen atoms are not displayed for better visualization. **(B)** The radiosynthesis of [^255^Ac]αMSH-PEG-Cy5-C′ dots uses a two-step labeling process. In Step 1 ^255^Ac radioisotope is first chelated by DOTA-NCS and in Step 2, [^255^Ac]DOTA-NCS is conjugated to primary amine functional groups on the αMSH peptide. **(C)** Actinium-225 decay scheme showing each ^225^Ac radionuclide decay yields an α particle as well as several α-emitting daughters. ^225^Ac, actinium-225; αMSH, α melanocyte-stimulating hormone.

A sol–gel silica synthetic approach in water as solvent and polyethylene glycol layer as shell yielded spherical, water-soluble ultrasmall fluorescent core–shell silica nanoparticles (C′ dots) with a narrow size distribution.^39,40^ These fluorescent core–shell silica nanoparticles have a 6.0 nm diameter and contained on average 1.3 Cy5 dyes and 7.0 αMSH per particle (Supplementary Fig. S1). Radiochemical labeling methods to produce [^225^Ac]αMSH-PEG-Cy5-C′ dots (Fig. 1A) and [^225^Ac]NH_2_-PEG-Cy5-C′ dots are based on a two-step labeling approach and are illustrated in Figure 1B. This radiochemical methodology has been designed to radiolabel temperature-sensitive proteins and subsequently translated and validated for clinical α immunotherapy in the treatment of leukemia using [^225^Ac]Lintuzumab.^14,16^ In Step 1, ^225^Ac nitrate (0.023 ± 0.014 GBq [mean ± standard deviation]; *n* = 8) is bound by the bifunctional DOTA-Bz-SCN (0.66 ± 0.27 mg; *n* = 8) chelate, which controls the pharmacokinetic fate of radionuclide dispersal *in vivo*^13^ and avoids nonspecific binding of radionuclide onto the C′ dot particle. This first reaction proceeds to 100% completion (*n* = 8) under these conditions. In Step 2, [^225^Ac]DOTA-Bz-SCN was reacted with the dLys epsilon amino group on the αMSH analog (Ac-Cys-(aminohexanoic acid)_2_-dLys-Re[Cys-Cys-Glu-His-dPhe-Arg-Trp-Cys]-Arg-Pro-Val-NH_2_) through the reactive isothiocyanate moiety. The resulting [^225^Ac]DOTA-Bz-SCN product was then added directly to αMSH-PEG-Cy5-C′ dot (2.8 ± 1.6 nmol; *n* = 6) and reacted for 42 ± 16 min (*n* = 6). Purified [^225^Ac]αMSH-PEG-Cy5-C′ dots were isolated using SEC and assayed for radiochemical purity at secular equilibrium (97.8% ± 2.0%; *n* = 6). The radiochemical yield of the second step was 2.9% ± 1.8% (*n* = 6). The specific activity was 236,115 ± 106,722 GBq/mol; the activity concentration was 1.53 ± 2.06 GBq/L; and the αMSH-PEG-Cy5-C′ dot concentration was 4.80 ± 5.77 μmol/L (all *n* = 6). The [^225^Ac]NH_2_-PEG-Cy5-C′ dot (hydrodynamic diameter of 6.6 nm and 1.4 Cy5 dyes per particle (Supplementary Fig. S1) used as a nonspecific control (3.0 ± 2.1 nmol of C′ dot, *n* = 2) was reacted for 35 ± 7 min and were 98.9% ± 0.21% radiochemically pure. The radiochemical yield of the second step was 7.5% ± 6.0% (*n* = 2). This C′ dot control had specific activity of 250,778 ± 40,698 GBq/mol; activity concentration 0.37 ± 0.29 GBq/L; and NH_2_-PEG-Cy5-C′ dot concentration of 1.42 ± 0.94 μmol/L (*n* = 2).

**FIG. 2.**
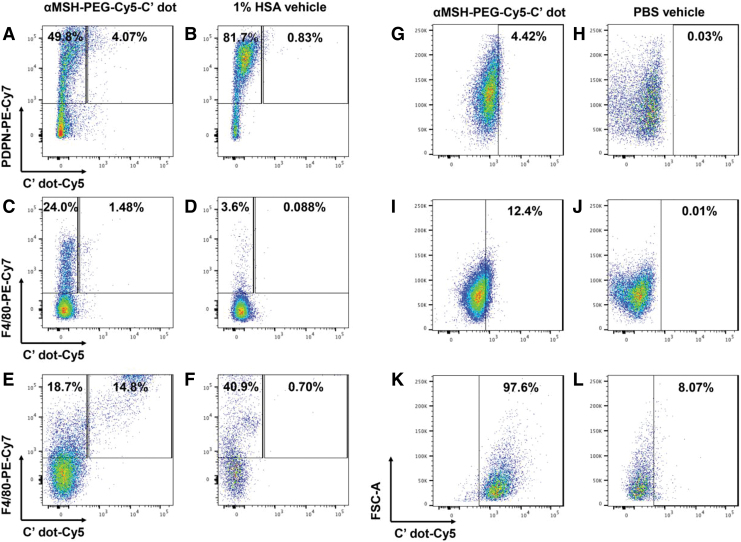
Internalization of αMSH-PEG-Cy5-C′ dots by B16-F10 tumor cells and macrophages. FACS plot of PDPN-PE-Cy7 versus C′ dot Cy5 in B16-F10 cells isolated from tumor 4 d after intravenous administration of **(A)** 50 pmol of αMSH-PEG-Cy5-C′ dots or **(B)** 1% HSA injection. FACS plot of F4/80-PE-Cy7 versus C′ dot Cy5 in macrophages isolated from tumor 4 d after intravenous administration of **(C)** 50 pmol of αMSH-PEG-Cy5-C′ dots or **(D)** 1% HSA injection. FACS plot of F4/80-PE-Cy7 versus C′ dot Cy5 in IP tissue macrophages harvested from naive mice 2 d after IP administration of **(E)** 50 pmol of αMSH-PEG-Cy5-C′ dots or **(F)** 1% HSA injection. FACS analysis of FSC versus C′ dot Cy5 at 2 d after introduction of **(G)** 25 pmol of αMSH-PEG-Cy5-C′ dots or **(H)** 1 × PBS addition to B16-F10 cells *in vitro*. FACS analysis of FSC versus C′ dot Cy5 at 2 d after introduction of **(I)** 25 pmol of αMSH-PEG-Cy5-C′ dots or **(J)** 1 × PBS addition to wild-type THP-1 cells *in vitro*. FACS analysis of FSC versus C′ dot Cy5 at 2 d after introduction of **(K)** 25 pmol of αMSH-PEG-Cy5-C′ dots or **(L)** 1 × PBS addition to PMA-differentiated THP-1 cells *in vitro*. FACS, fluorescence-activated cell sorting; FSC, forward scatter; HSA, human serum albumin; IP, intraperitoneal; PBS, phosphate-buffered saline; PMA, phorbol 12-myristate 13-acetate.

Flow cytometry investigations of tumor cells and macrophages confirmed that both cell types internalized αMSH-PEG-Cy5-C′ dots *in vivo* and *in vitro*. Intravenously administered αMSH-PEG-Cy5-C′ dots in mice with B16-F10 melanoma showed accumulation of the silica nanoparticles in both PDPN^+^ melanoma cells (4.07%) and F4/80^+^ macrophages (1.48%) (Fig. 2A–D). αMSH-PEG-Cy5-C′ dots administered intraperioneally to naive mice were also found to localize in the IP tissue macrophage population (14.8%) versus vehicle (0.70%) (Fig. 2E, F). FACS analyses for both these experiments *in vivo* used the 1% HSA vehicle (containing no αMSH-PEG-Cy5-C′ dots) as a control. Tissue culture experiments also established that αMSH-PEG-Cy5-C′ dots were internalized by B16-F10 cells (4.42%), wild-type THP-1 cells (12.4%), and PMA-differentiated THP-1 cells (97.6%) at 48 h (Fig. 2G–L). FACS analyses of experiments *in vitro* used the PBS vehicle (no αMSH-PEG-Cy5-C′ dots) as a control. Experiments *in vitro* indicated slower uptake kinetics where less nanoparticle was internalized at 1 d than 2 d. C′ dot internalization plateaued at 2 d with only minimal additional accumulation at 3 and 4 d time points in the B16-F10, THP-1, and PMA-differentiated THP-1 cells.

**FIG. 3.**
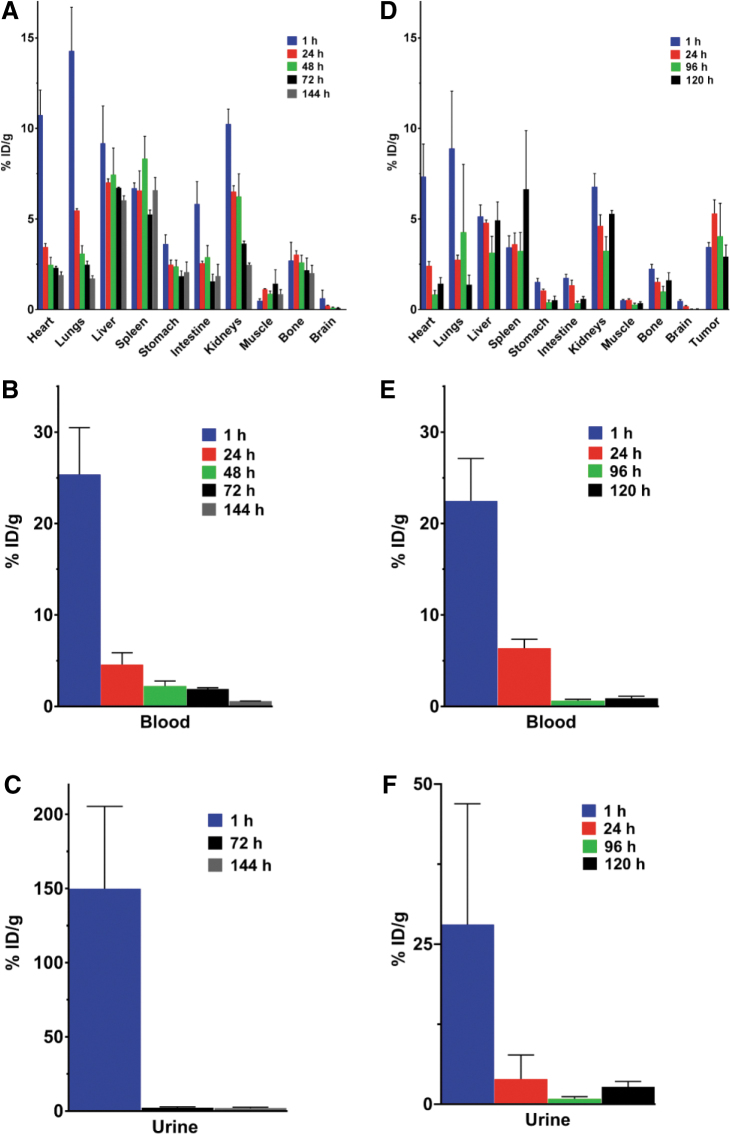
Pharmacokinetic profile of [^225^Ac]αMSH-PEG-Cy5-C′ dots in naive and B16-F10 tumor-bearing C57BL/6J mice. **(A)** Tissue biodistribution, **(B)** blood clearance, and **(C)** urinary excretion of [^225^Ac]αMSH-PEG-Cy5-C′ dots in healthy naive mice (*n* = 3 per group) at 1, 24, 48, 72, and 144 h postinjection expressed as %ID per gram of tissue. **(D)** Tissue biodistribution, **(E)** blood clearance, and **(F)** urinary excretion of [^225^Ac]αMSH-PEG-Cy5-C′ dots in tumor-bearing mice (*n* = 5 per group) at 1, 24, 96, and 120 h postinjection expressed as %ID per gram of tissue. Data are reported as the mean ± SEM. SEM, standard error of the mean.

Pharmacokinetic data describing tissue biodistribution, blood clearance, and renal elimination of [^225^Ac]αMSH-PEG-Cy5-C′ dots in healthy naive animals are shown in Figure 3A–C. ^225^Ac activity in the blood compartment dominates the pharmacokinetic profile at early time points (25.37 ± 8.87%ID/g at 1 h postinjection; *n* = 3) and is accompanied by rapid renal clearance (149.9 ± 96.1%ID/g at 1 h; *n* = 3) of the ultrasmall silica particles. Blood activity decreases during the first day *in vivo* to 4.59 ± 2.24%ID/g (*n* = 3) at 24 h postinjection, and further urinary excretion is minimal (<2.5%ID/g). The sum of the mean %ID that accumulated in all harvested tissues from each animal (*n* = 10) is 11.12 ± 1.58 and there is on average only 1.11 ± 0.12%ID per tissue. Liver, spleen, and kidney have the greatest accumulation of nanoparticles (7.02 ± 0.35, 6.58 ± 1.86 and 6.52 ± 0.54%ID/g, respectively).

Parallel pharmacokinetic analyses of [^225^Ac]αMSH-PEG-Cy5-C′ dot tissue biodistribution, blood clearance, and renal elimination in syngeneic melanoma engrafted mice is shown in Figure 3D–F. Again, the blood compartment activity dominates the pharmacokinetic profile at early time points (22.47 ± 10.39%ID/g at 1 h postinjection; *n* = 5) and is accompanied by the rapid renal clearance (28.07 ± 42.15%ID/g at 1 h; *n* = 5) of untargeted C′ Dots. Blood activity decreases to 6.37 ± 2.19%ID/g; *n* = 5) at 24 h postinjection and further urinary excretion of the C′ dots is low (<4%ID/g). The intravenously administered activity exhibits biphasic elimination kinetics with a Phase 1 effective half-life of 0.46 d and Phase 2 half-life of 8.1 d. Tumor accumulates 5.30 ± 1.71%ID/g (*n* = 5) of the injected activity at 1 d and the retention has an effective half-life of 115.5 h. The sum of the mean %ID that accumulated in all harvested tissues from each mouse (*n* = 10), not including tumor, is 7.79 ± 1.25 and there is on average only 0.78 ± 1.25%ID per tissue. Liver has the greatest accumulation of nanoparticle (4.79 ± 0.36%ID/g) and spleen and kidney have 3.61 ± 1.38 and 4.62 ± 1.38%ID/g, respectively.

**FIG. 4.**
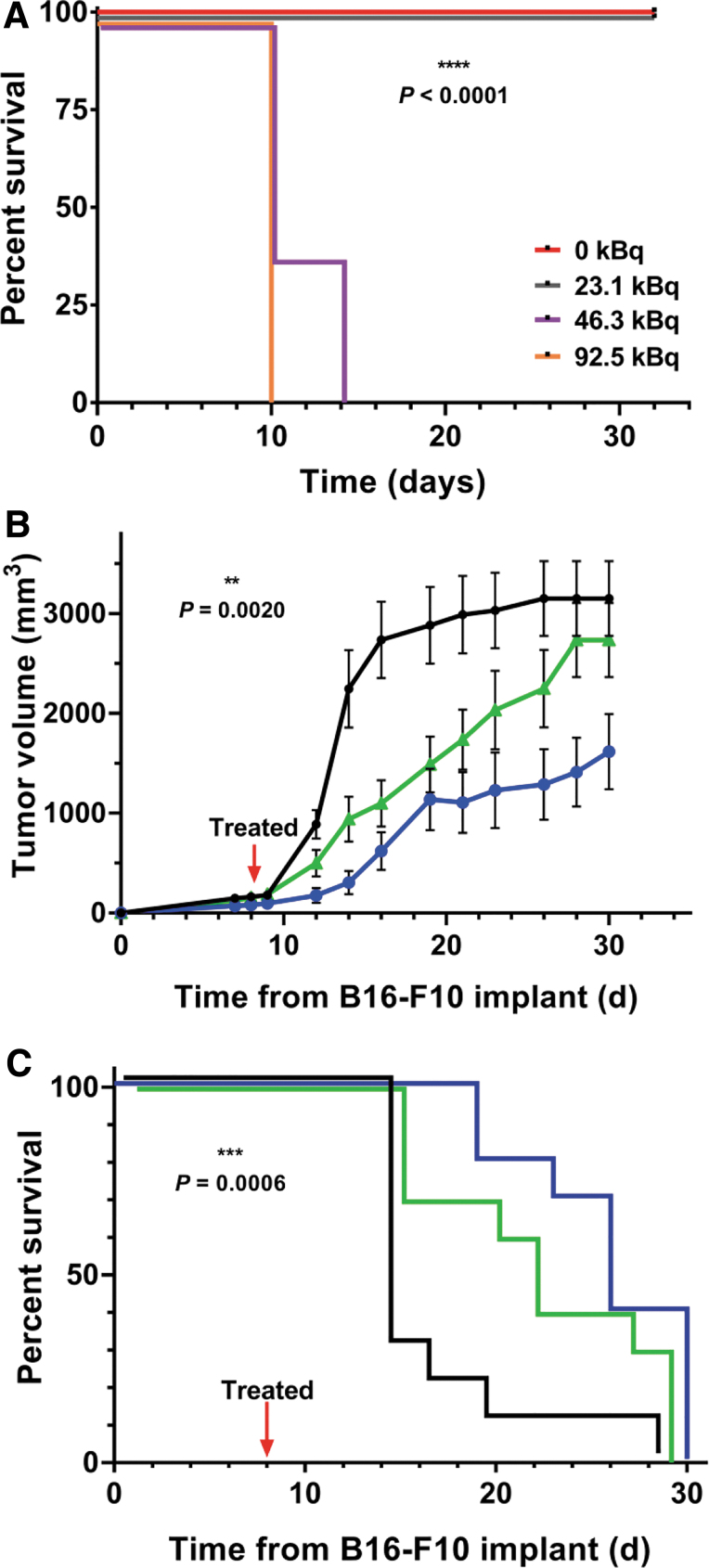
Pharmacodynamic profile of [^225^Ac]αMSH-PEG-Cy5-C′ dots bioactivity in naive and syngeneic B16-F10 tumor-bearing C57BL/6J mice. **(A)** Determination of the maximum tolerated dose of [^225^Ac]αMSH-PEG-Cy5-C′ dots in naive C57BL/6J mice (*n* = 5 per group) that received 0 (*red line*), 23.1 (*gray line*), 46.3 (*purple line*), or 92.5 kBq (*orange line*) per mouse. The *curves* are nudged to separate overlaying data for better visualization. Alpha particle radiotherapeutic effects on B16-F10 **(B)** tumor volume and **(C)** survival in C57BL/6J mice following a single intravenous dose of 11.1 kBq and 55 pmol of targeted [^225^Ac]αMSH-PEG-Cy5-C′ dot (*blue*); 11.1 kBq and 55 pmol of nontargeted [^225^Ac]NH_2_-PEG-Cy5-C′ dot (*green*), or the 1% HSA injection vehicle (*black*). All three group sizes are *n* = 10. The *curves* are nudged to separate overlaying data. Data are mean ± SEM in **(B)**. HSA, human serum albumin.

The [^225^Ac]αMSH-PEG-Cy5-C′ dot absorbed dose to tumor is estimated to be 2412 cGy/MBq. The normal organ-absorbed doses (Table 1) ranged from only a few rads to a few tens of rads for the administered activity of 11.1 kBq of [^225^Ac]αMSH-PEG-Cy5-C′ dots and correlates with the observation that there was no pronounced normal tissue toxicity in the pharmacokinetic or pharmacodynamic studies. Additional data for dose estimates to the normal organs in mice and to normal organs in the 70-kg Reference Man are presented in Table 1. The mouse absorbed doses on a per-MBq basis are, of course, much higher than the Reference Man dose, reflecting the orders of magnitude difference in body mass between mouse and man. In man, the organ-absorbed doses are uniformly of the order of 1 cGy/MBq, except for 11.4 cGy/MBq delivered to the kidneys. The maximum tolerated dose (MTD) of [^225^Ac]αMSH-PEG-Cy5-C′ dot was at least 23.1 kBq (0.63 μCi per mouse) and below 46.3 kBq (1.26 μCi per mouse) in healthy, naive mice (Fig. 4A). Median survival was undefined in the groups that received 0 or 23.1 kBq and 10 d in mice that received either 46.3 or 92.5 kBq. Human dosimetry predictions (70-kg man) for a 37 MBq dose of [^225^Ac]αMSH-PEG-Cy5-C′ dot predicted that the absorbed dose to kidney, liver, and lung are 4.2, 1.9, and 0.99 Gy, respectively, and these are significantly below the dose limits of 23, 40, and 20 Gy for these organs, respectively.

**Table 1.** [^225^Ac]αMSH-PEG-Cy5-C′ Dot Absorbed Doses in Mice and in the 70-kg Reference Man

Tissue	Absorbed dose, cGy/MBq	
	Mouse	Ref. Man
Brain	45	0.773
Large intestine	472	2.23
Stomach wall	1.55	
Heart wall	877	1.80
Kidneys	4792	11.4
Liver	1275	5.22
Lungs	1574	2.68
Muscle	240	0.757
Red marrow	1.25	
Bone	1290	60.0
Spleen	1275	4.41
Total body	2.15	
Tumor	2412	

Pharmacodynamic studies examined B16-F10 tumor control, host survival, and associated effects on the TME immune cell content using an immunocompetent mouse model of melanoma following a single IV administration of 11.1 kBq (300 nCi) of [^225^Ac]αMSH-PEG-Cy5-C′ dots. Control experiments included the injection of vehicle as a growth control and a nonspecific [^225^Ac]NH_2_-PEG-Cy5-C′ dot particle. This therapy study employed a radioactivity dose ∼50% lower than MTD to mitigate nonspecific effects.^13^ Tumor volumes were measured longitudinally and presented in Figure 4B. Linear tumor growth becomes exponential at ∼10 d postimplantation in the vehicle-treated growth control group. Nonspecific radiation effects arising from the nontargeting particle delay the rate of tumor growth compared with the growth control. Specific tumor growth control is observed with a decrease of >50% tumor volume when compared with the vehicle group on day 30. Separation in the tumor volume curves is observed between the specific and nonspecific groups throughout the course of the study. Kaplan–Meier analysis reports median survival times of 14, 21, and 26 d for the vehicle, nonspecific, and specific groups, respectively (Fig. 4C). A Log-rank (Mantel–Cox) test shows a statistically significant difference (*p* = 0.0020) in the survival data for all three groups. Comparison of the specific group with the vehicle control is statistically significant (*p* = 0.0006) and a Hazard Ratio of 9.986 (95% confidence interval is 2.671–37.33) using the Mantel–Haenszel test.

**FIG. 5.**
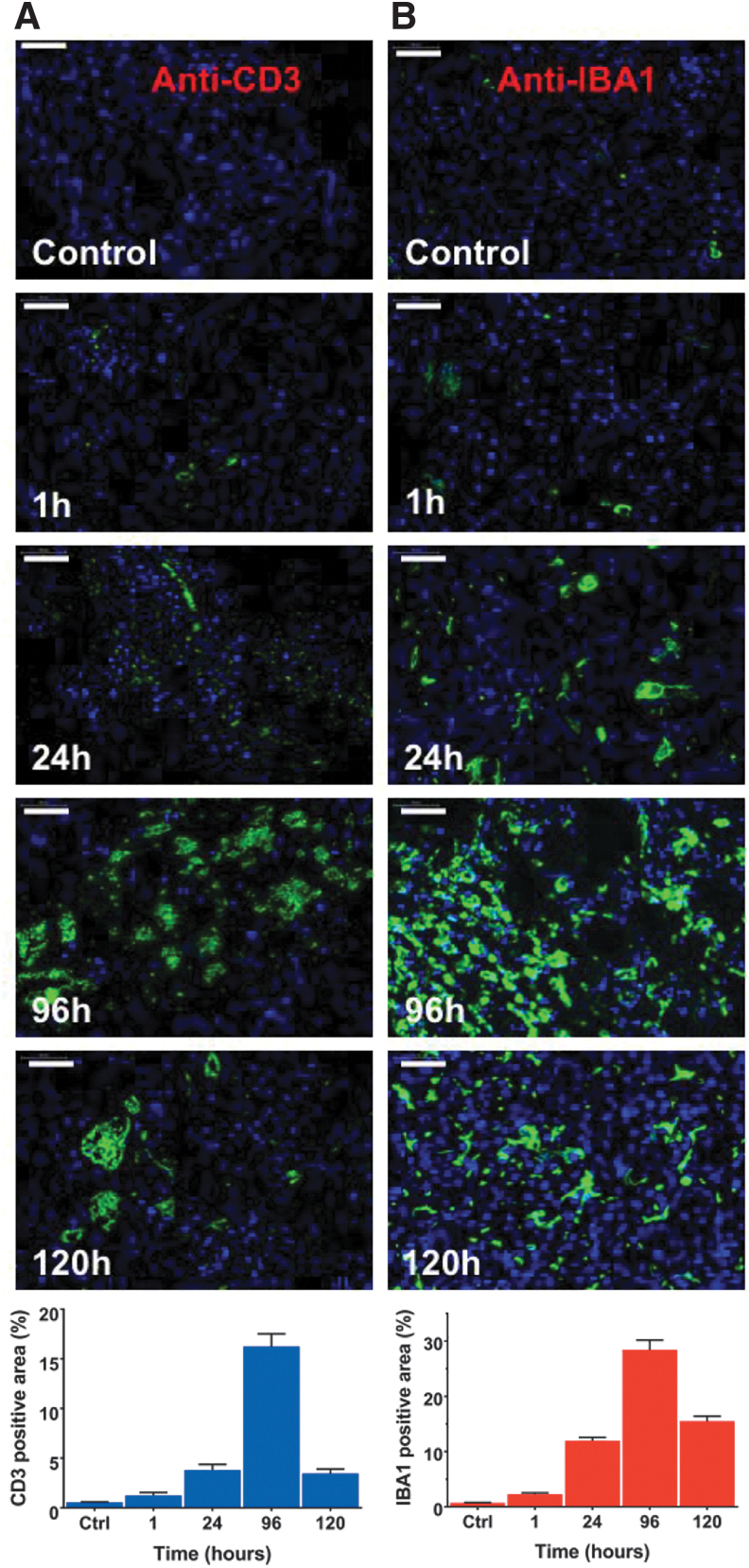
Immune cell changes in the syngeneic tumor microenvironment following treatment with [^225^Ac]αMSH-PEG-Cy5-C′ dots. Representative images of immune cells in the B16-F10 tumor microenvironment and quantification of signal (mean ± SEM). Tumor tissue was harvested at 1, 24, 96, and 120 h post-treatment and stained with **(A)** anti-CD3 and **(B)** anti-IBA1 immunofluorescence markers to identify time-dependent changes in T cell and macrophage populations, respectively. These tumor samples were obtained from the animals in the pharmacodynamic therapy study. Images of untreated tumor tissue are included as control (Ctrl). Immunofluorescence stains of T cells (*green*) and macrophages (*green*) counterstained with DAPI (*blue*). Scale bars are 50 μm. DAPI, 4′,6-diamidino-2-phenylindole.

Immune cells populating the α-irradiated TME were characterized using IF staining of tumor harvested at different times after [^225^Ac]αMSH-PEG-Cy5-C′ dot treatment. Distinct changes in immune phenotypes were observed as a function of time from treatment (Fig. 5). Anti-CD3 and anti-IBA1 staining shows time-dependent increase in T cells (at 96 h the anti-CD3 signal is 30-fold greater than baseline) and macrophage cells (at 96 h the anti-IBA1 signal is 40-fold greater than baseline) in the TME. Image quantification demonstrates that T cell (CD3^+^) and macrophage (IBA1^+^) expression peak 4 d following treatment.

**FIG. 6.**
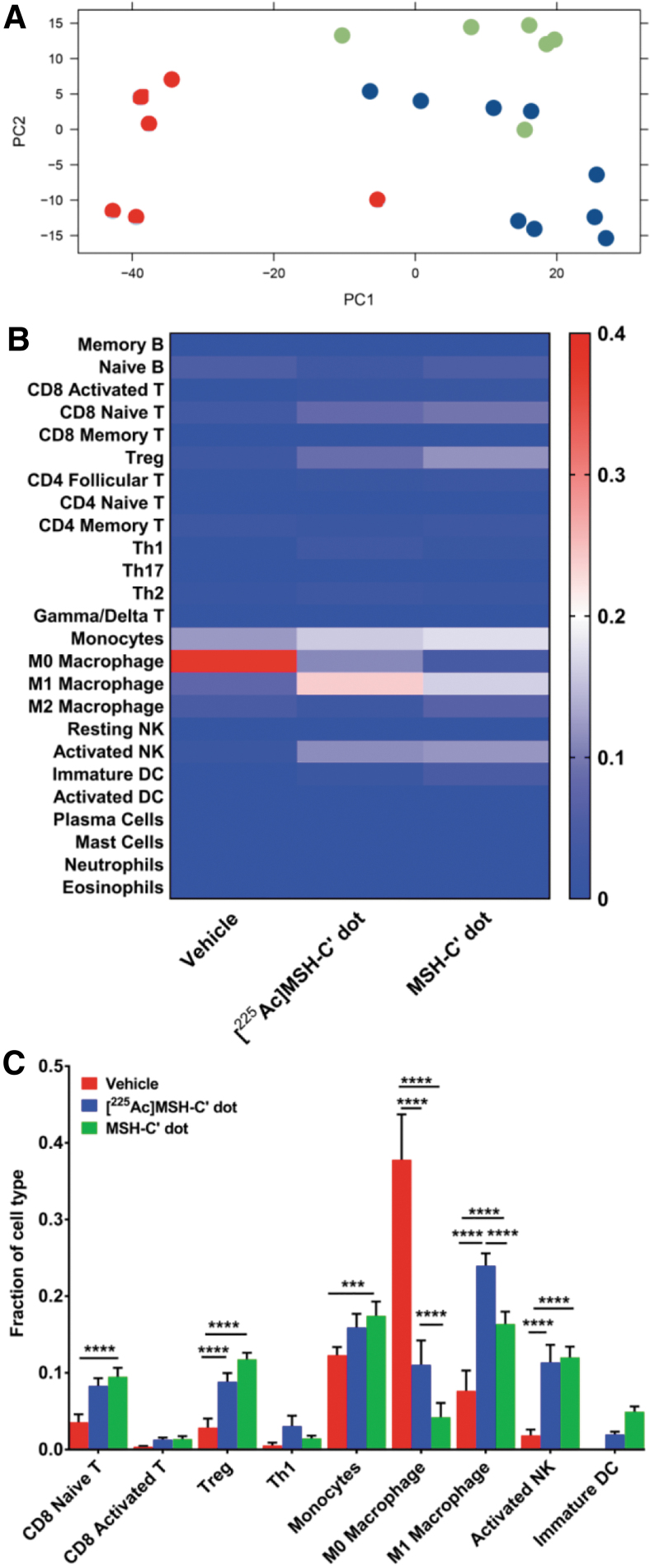
Immune cell composition of the tumor microenvironment after treatment with [^225^Ac]αMSH-PEG-Cy5-C′ dots or unlabeled αMSH-PEG-Cy5-C′ dots. **(A)** An unsupervised PCA showing the first two principal components of all samples using data obtained from RNA-seq of a vehicle-treated control group that received only vehicle (*red*), an [^225^Ac]αMSH-PEG-Cy5-C′ dot-treated group (*blue*), and an unlabeled αMSH-PEG-Cy5-C′ dot-treated control group (*green*). **(B)** A heat map of the mean fraction of immune cell signatures in the CD45^+^ cells isolated from individual B16-F10 tumors calculated using CIBERSORT and ImmuneCC algorithms. **(C)** Analysis of populational changes in T cells, macrophages, monocytes, and natural killer cells within the tumor microenvironment as a function of treatment. PCA, principal component analysis.

Transcriptome sequencing of all CD45-positive cells isolated from “hot” [^225^Ac]αMSH-PEG-Cy5-C′ dot- and “cold” αMSH-PEG-Cy5-C′ dot-treated tumors (and vehicle-treated controls) provided an extensive gene dataset to analyze the immune cell signatures in the TME at 96 h post-treatment. This time point was selected based on the results of the IF experiments where maximal changes in T cell and macrophage numbers were observed in the TME versus untreated growth controls. Computational interrogation of differentially expressed genes in each group versus controls yielded heatmaps (Supplementary Fig. S2A–C) indicating patterns of up- and downregulated genes. An unsupervised principal component analysis of these data (Fig. 6A) also demonstrated distinct treatment-based effects for both the radiolabeled and unlabeled targeted C′ dots relative to the vehicle-treated controls. These data were then evaluated to infer the relative fractions of immune cells in each tumor (Fig. 6B, C and Supplementary Fig. S3) using CIBERSORT and ImmuneCC algorithms. Heat maps demonstrate important population shifts as a function of treatment and statistical analyses report significant increases in naive CD8 T cells, T regulatory (Treg) cells, monocytes, MΦ and M1 macrophages, and activated natural killer (NK) cells arising from either the ^225^Ac-labeled or unlabeled αMSH-PEG-Cy5-C′ dots compared with the vehicle-treated tumors (Fig. 6B, C).

Innate immunity changes in the TME entail increases in the fraction of classically activated macrophages (M1) for both nanoparticle treatment groups (“hot” radiolabeled C′ dot is 0.2397 ± 0.0486 [*n* = 9] and “cold” unlabeled C′ dot is 0.1636 ± 0.0397 [*n* = 6]) versus vehicle-treated controls (0.0766 ± 0.0648 [*n* = 6]). The TME monocyte content increased in the hot (0.1592 ± 0.05317) and cold (0.1744 ± 0.04579) treated groups relative to untreated controls (0.1231 ± 0.02594). Infiltration of activated NK cells increases in hot (0.1137 ± 0.0686) and cold C′ dot-treated tumors (0.1200 ± 0.0346) versus vehicle-treated controls (0.0183 ± 0.0184). The fraction of MΦ macrophages decreased significantly following treatment with either hot (0.1105 ± 0.09537) or cold (0.0421 ± 0.04568) targeted C′ dot treatment versus untreated controls (0.378 ± 0.1448). Immature dendritic cells (DC) were not detected in vehicle-treated tumors but the fractions of these antigen-presenting cells increased in the hot (0.0120 ± 0.0111) and cold (0.0493 ± 0.0168) treated groups.

The adaptive immune response is also engaged, and the fraction of activated CD8 T cells increased several fold after both hot (0.0129 ± 0.0082) and cold C′ dot treatment (0.0136 ± 0.0091) relative to the vehicle-treated control groups (0.0036 ± 0.0026). The fraction of naive CD8 T cells increased following hot (0.08286 ± 0.03066) and cold C′ dot treatment (0.09497 ± 0.02825) versus vehicle-treated controls (0.03557 ± 0.02508). Similarly, the fraction of Th1 cells increased in hot (0.0307 ± 0.0328) and cold (0.0144 ± 0.0085) treated animals versus untreated controls (0.0054 ± 0.0090). Interestingly, the number of Treg cells also increased in the hot (0.0884 ± 0.0341) and cold (0.1176 ± 0.02088) treated animals versus the untreated controls (0.02852 ± 0.02895).

While the α particle radiotherapy study showed specific and potent tumor control derived from [^225^Ac]αMSH-PEG-Cy5-C′ dots (Fig. 4B, C), an additional therapy study was included to investigate tumor control arising from a single administration of 55 pmol of unlabeled cold αMSH-PEG-Cy5-C′ dots on day 8 versus vehicle-treated controls (Supplementary Fig. S4). The unlabeled cold C′ dots are not as cytotoxic nor as effective in controlling tumor growth as the hot radiolabeled C′ dots (Supplementary Fig. S4A, B). Only a slight delay in tumor growth is noted at 18 d versus the untreated controls. Kaplan–Meier analysis reports median survival times of 18 and 25 d for the vehicle and cold C′ dot groups, respectively (Supplementary Fig. S4B). A Log-rank (Mantel–Cox) test shows a statistically significant difference (*p* = 0.0341) in the survival data for these two groups. However, the transcriptome analysis clearly indicates that the cold targeted particle does exert an effect on the immune cells populating the TME. It is important to note that the hot and cold targeted C′ dots have comparable immune cell fractions compared with the vehicle-treated tumors, inferring that the C′ dot platform alone has a dominant role in TME local immunity. While it is evident that both labeled and unlabeled αMSH-PEG-Cy5-C′ dots prompt changes in CD8 T and Treg cells, monocytes, MΦ and M1 macrophages, and activated NK cells, the cytotoxic ^225^Ac component of the drug is necessary to introduce a potently cytotoxic element and effect tumor control.

An analysis of cytokine gene expression in these three groups indicated that both the hot and cold αMSH-PEG-Cy5-C′ dots yield similar profiles in the TMEs CD45^+^ immune cells versus the vehicle-treated controls (Supplementary Fig. S5 and Supplementary Table S1). The expression of several granzyme genes (*Gzma, Gzmb, Gzmc, Gzmd, Gzme, Gzmf,* and *Gzmg*) was particularly robust in both hot and cold-treated groups and ranged from 5- to 28-fold higher expression compared with the vehicle-treated group. Other inflammatory cytokines and receptors identified in this analysis include the interleukins (*Il12rb1, Il18bp*, *Il2rb, Il27*), interferon γ (*Ifng*), interferon-induced proteins (*Ifit1, Ifit1bl1, Ifit2, Ifit3, Ifit3b*), tumor necrosis factor (TNF) ligand family (*Tnfsf10, Tnfsf11, Tnfsf13b, Tnfsf14, Tnfsf15, Tnfsf4, Tnfsf8*), and chemokine (C-C motif) ligands (*Ccl1, Ccl11, Ccl17, Ccl22, Ccl4, Ccl5, Ccl8*). The direct comparison of gene expression between the hot and cold groups does not demonstrate remarkable differences in cytokine-related expression.

Pathway enrichment analysis of differentially expressed genes with at least fourfold change demonstrated that many of the top upregulated pathways in “hot” C′ dot-treated tumors versus vehicle-treated controls are immunity, immune response, adaptive immunity, cellular response to interferons, and response to virus (Supplementary Table S2). This analysis infers that pathways that control cytolysis, peptidase, protease, proteolysis, apoptotic response, hydrolase activity, and viral response, among others, are upregulated in the CD45^+^ cells that populate C′ dot-treated TME.

## Discussion

Ultrasmall silica nanoparticles with fluorescent core–shells have been engineered to display a unique combination of biochemical features enabling them to target and treat melanoma *in vivo*. The targeted α particle-emitting ^225^Ac payload enables a potent and specific tumoricidal effect that controls tumor growth at doses that are safe and nontoxic to normal tissue. These same nanoparticles are internalized by macrophages and unexpectedly, even the unlabeled targeted particles alone are sufficient to prompt key inflammatory immune cell changes within the TME. Pharmacologically, αMSH-functionalized C′ dots target melanoma, clear the host rapidly, deliver therapeutic payloads of cytotoxic α particles to disease, and significantly alter the immune cell composition within the TME through macrophage processing and inflammatory signaling.

The overall pharmacokinetic profile of [^225^Ac]αMSH-PEG-Cy5-C′ dots is governed by the ultrasmall silica particle size and shape in both naive and melanoma-bearing mice, where the αMSH permits tumor-specific binding and internalization. Actinium-225 activity clears the blood compartment with biphasic elimination kinetics in both models. Due to the 6.0 nm diameter of these ultrasmall particles, C′ dots are readily eliminated in urine in both naive and tumor-bearing mice. Rapid renal elimination of C dots was also noted in humans and is a favorable pharmacological characteristic in translation.^5^ Specific tumor accumulation, minimal off-target tissue uptake, rapid clearance from blood, and facile renal elimination are all well suited for both therapeutic and diagnostic medical applications in humans. Additional new data presented in this study establishes that macrophages in naive and tumor-bearing mice are also a sink for the αMSH-PEG-Cy5-C′ dots *in vivo*. Macrophage uptake of the targeted silica nanoparticles is related to key changes in the immune cell profile of the TME.

Dose selection for therapeutic studies was informed from an evaluation of the drug's MTD. Naive mice receiving 23.1 kBq (625 nCi) of [^225^Ac]αMSH-PEG-Cy5-C′ dots exhibit no toxicity (i.e., there was less than 20% weight loss and no lethargy or death at this dose level) and median survival was not reached. This absence of radiobiological effects on the animal's health can be explained by the favorable pharmacokinetic characteristics of the radiolabeled αMSH-C′ dot as it does not significantly accumulate in normal tissue and untargeted drug is rapidly eliminated from the host. Higher dose levels of ^225^Ac-labeled C′ dots (46.3 or 92.5 kBq per mouse) were toxic and median survival was 10 d. Radiotherapeutic studies used approximately half the MTD (11.1 kBq, 300 nCi) to avoid nonspecific effects as previously described.^13^

Potent and specific pharmacodynamic activity was observed in a syngeneic melanoma mouse model. A single 11.1 kBq dose of [^225^Ac]αMSH-PEG-Cy5-C′ dot effectually controls tumor growth and improves survival compared with a nontargeted [^225^Ac]NH_2_-PEG-Cy5-C′ dot control and vehicle-treated groups. Tumor-specific [^225^Ac]αMSH-PEG-Cy5-C′ dot improves median survival compared with vehicle-treated mice. Specific tumor control is evidenced in the separation between the mean tumor volumes of specific and nonspecific C′ dot-treated groups over the course of the study. Human dosimetry predictions for a 37 MBq dose of [^225^Ac]αMSH-PEG-Cy5-C′ dot project that absorbed doses to kidney, liver, and lung are significantly below the dose limits for these organs.

Tumor control and immune cell changes in the TME indicate potent cytotoxicity and a dynamic, time-dependent remodeling of the immune phenotype following [^225^Ac]αMSH-PEG-Cy5-C′ dot treatment compared with vehicle-treated control animals. The direct pharmacological consequences of α particle irradiation and the silica nanoparticle contribute to tumor killing and TME remodeling. Dynamic changes in macrophage, T cell, and NK cell populations were observed over a 4–5 d period and suggest that ancillary immunotherapeutic approaches^41–46^ be deployed in combination with the ^225^Ac-labeled C′ dot agents. RNA-seq was used to identify specific immune cell signatures in the TME that occur 4 d after treatment. Surprisingly, the cold αMSH-PEG-Cy5-C′ dots also induced comparable changes in the TME that are similar to the hot [^225^Ac]αMSH-PEG-Cy5-C′ dots. However, the radiolabeled C′ dot drug was more immediately cytotoxic than the cold C′ dot as it potently reduced tumor burden, thus improving overall survival. An unsupervised principal component analysis of gene expression from all samples showed overlap in both C′ dot-treated groups (i.e., labeled and unlabeled), which were distinct from the vehicle-treated controls.

**FIG. 7.**
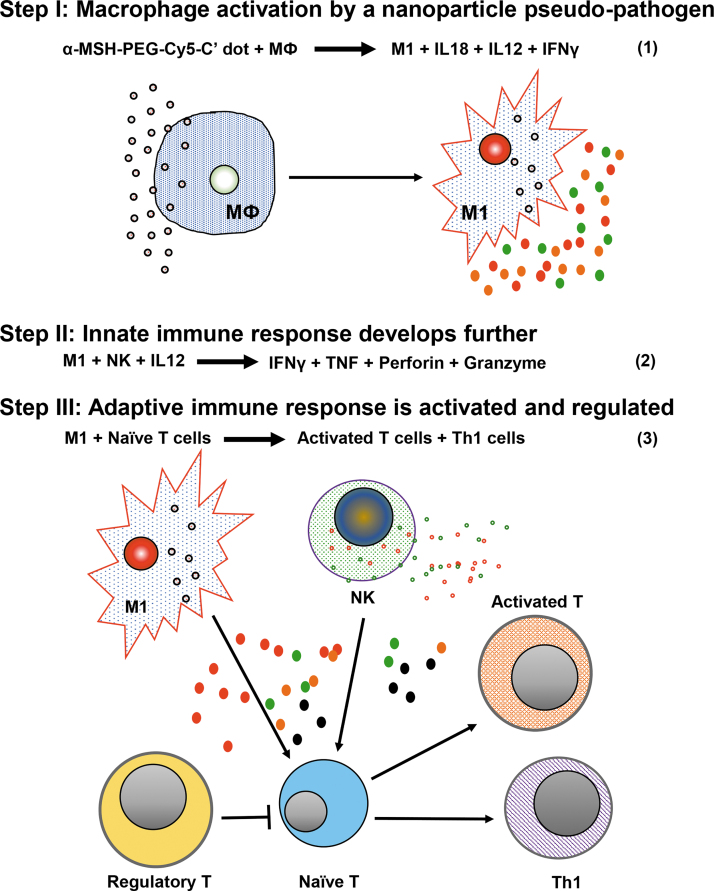
Proposed mechanism of action for a macrophage-initiated, pseudopathogenic response to αMSH-PEG-Cy5-C′ dots in the tumor microenvironment.

FACS analyses demonstrated that αMSH-PEG-Cy5-C′ dots were internalized by both B16-F10 melanoma and macrophages. When radiolabeled with ^225^Ac, the accumulation of C′ dots in tumor yields the optimal geometry for specific cytotoxic α particle irradiation of the melonoma. When C′ dots are taken up by macrophages, they cue a dynamic immunoreactive environment within melanoma that engages both innate and adaptive response elements. We observe distinct increases in monocytes and M1 macrophages in the TME as well as increased naive and activated CD8 T, Th1, activated NK cells, and immature DC. The fraction of Treg cells also increases in the treated TME, potentially suppressing favorable immunotherapeutic tumor responses. We have also observed αMSH-PEG-Cy5-C′ dot uptake in murine IP tissue macrophages *in vivo*. Furthermore, human THP-1 cells (wild type and PMA differentiated) and B16-F10 also accumulated αMSH-PEG-Cy5-C′ dots *in vitro*. Activated THP-1 cells are reported to express MC1R^47^ and these data show that macrophages phagocytose and accumulate αMSH-PEG-Cy5-C′ dots. Mechanistically, we hypothesize that the ultrasmall silica dots are phagocytosed by macrophages prompting a pseudopathogen immunologic response (Fig. 7). This early innate immune response subsequently engages and activates and expands the relative numbers of NK, Th1, CD8 T, and immature DC (Table 2). Upregulated cytokine and cytolytic protein gene expression is additional evidence that numerous key inflammatory signals increase in the TME as a consequence of C′ dot macrophage pharmacology (Table 2). Furthermore, the expression of granzymes, interleukins, interferon γ, interferon-induced proteins, TNF ligands, and chemokines describe a complex milieu of inflammatory signaling molecules arising from the C′ dot component of the drug. Upregulated pathways in [^225^Ac]αMSH-PEG-Cy5-C′ dot-treated tumors versus vehicle-treated control are immunity, immune response, adaptive immunity, and cellular response to interferons and are consistent with response to a viral pathogen.^48^

**Table 2.** Fold Changes in Representative Immune Cells and Cytokines and Cytolytic Protein Gene Expression Levels Found in the Pseudopathogen-Activated Microenvironment 96 H After Treating Melanoma with Hot [^225^Ac]αMSH-PEG-Cy5-C′ Dots or Cold αMSH-PEG-Cy5-C′ Dots Versus Vehicle-Treated Controls

	Ratio of hot-to-control	Ratio of cold-to-control
Cell type		
MΦ macrophage	0.29	0.11
M1 macrophage	3.1	2.1
NK cell (activated)	6.0	6.3
CD8 T cell (naive)	2.3	2.6
CD8 T cell (activated)	3.3	3.3
Th1 cell	6.2	3.0
Regulatory T cell	3.0	4.1
Dendritic cell (immature)	≫2	≫5
Cytokines and cytolytic proteins		
IL18	6.8	5.0
IL12	8.9	8.6
IFNγ	7.6	8.5
TNF	3.6	3.4
Perforin	ND	ND
Granzyme	17.8	28.3

The C′ dot component of the drug undoubtedly prompts inflammatory changes in the TME, which is an active area of ongoing investigation looking to introduce an immunotherapeutic approach to eradicating residual disease.^41–46^ Importantly, the C′ dots are a synthetic nanoscale particle and not a live pathogen, therefore, this initial phenotype response has a finite lifetime *in vivo* and is not a self-sustaining event. We expect that the observed increase in TME Tregs dampens the tumoricidal immunologic activity, therefore, a goal moving forward is to uncover a means to maintain the initial antitumor immune response that we observe. The fraction of suppressive regulatory T cells in the TME increases in both the hot and cold-targeted C′ dot-treated groups increasing several-fold over baseline values in vehicle-treated tumor and recommends examination of anti-PD1 or anti-CTLA-4 checkpoint blockade strategies.^41,42^ Also, the CD47-SIRPα signaling axes in macrophages could be exploited to improve long-term tumor control.^43,44^ Finally, tumor killing from activated NK cells could be intensified with the introduction of IL12 or IFNγ.^45,46^ Another strategy entails administering only the “cold” MC1R-targeted C′ dots to initiate and sustain the pseudopathogenic response and remains an active area of investigation.

## Conclusions

Second-generation ultrasmall fluorescent core–shell silica nanoparticles have been designed to target melanoma *in vivo* through αMSH peptide moieties and produce potent and specific cytotoxicity due to an ^225^Ac payload. The pharmacologically active C′ dot agent is colloidally stable in aqueous solutions, biocompatible, and exhibits a narrow size distribution. A therapeutic α particle payload imparts cytotoxic high linear energy therapy and has been shown to be clinically beneficial in treating leukemia when delivered by Lintuzumab.^18,19^ Surprisingly, both radiolabeled [^225^Ac]αMSH-PEG-Cy5-C′ (“hot”) and unlabeled αMSH-PEG-Cy5-C′ dots (cold) similarly cue significant changes in the TME immune cell signatures. The inflammatory milieu is hypothesized to result from a pseudopathogenic response of macrophages to the C′ dot. This immune response upregulates the fraction of M1 macrophages, Th1, monocytes, activated NK, and immature DC in TME. Inflammatory pathways are engaged by this immune cell composite yielding a cytokine milieu that provides a distinctive opportunity to augment α therapy with ancillary immunotherapeutic approaches moving forward.

Melanoma is a heterogenous disease that is generally resistant to conventional radiotherapy. However, the cancer cells and TME may respond favorably to pharmacologic pressure applied from the targeted α particle therapy and a redirected host immune system.^21,23,27^ We show that a novel α particle emitting C′ dot that recognizes melanoma *in vivo* and delivers cytotoxic radiotherapy to disease also stimulates the infiltration and activation of several critical immune cell types. This information will be used to augment primary α therapy and potentially add durable immunological responses to the cytotoxic radiation event. Moving forward, the authors' program will investigate α-emitting C′ dots and adjunctive immunological and cytokine therapies in combinations that eradicate melanoma by activation of the host immune system to surveil and eliminate remaining cancer cells. This ultrasmall silica-based drug platform is a nanoscale material with favorable performance shown in animal models of disease; an analogous [^124^I]cRGDY-PEG-C′ dot particle was already translated into a Phase 1 first-in-human clinical trial to PET image metastatic melanoma,^5^ demonstrating the utility of these materials.

## Supplementary Material

Supplemental data

Supplemental data

Supplemental data

Supplemental data

Supplemental data

Supplemental data

Supplemental data
