# Supplementary material for: A Genomic Profile of Local Immunity in the Melanoma Microenvironment Following Treatment with α Particle-Emitting Ultrasmall Silica Nanoparticles
Source: Cancer Biother Radiopharm. 2020 Aug 13;35(6):459–73. doi: 10.1089/cbr.2019.3150 (PMC7462037; doi:10.1089/cbr.2019.3150)
Supplement: Supplemental data [file Supp_Fig1.pdf]

Supplementary Data

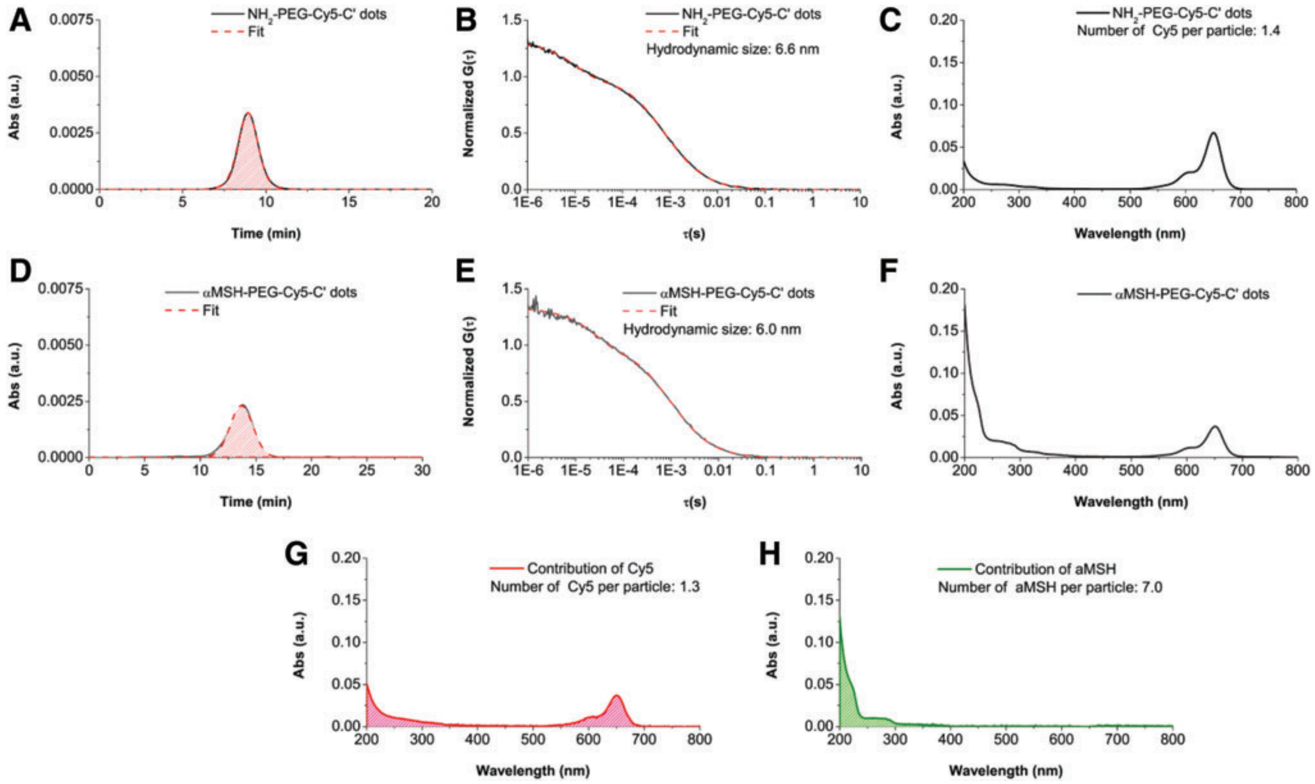

SUPPLEMENTARY FIG. S1. Characterization data for NH_2-PEG-Cy5-C′ dots and αMSH-PEG-Cy5-C′ dots. **(A, D)** GPG elugrams of C′ dots with fits (n.b., absolute GPC elution times are not comparable since these chromatograms were taken on different days using different columns); **(B, E)** FCS curves with fits and **(C, F)** UV-Vis absorbance of NH_2-PEG-Cy5-C′ dots and αMSH-PEG-Cy5-C′ dots, respectively. UV-Vis spectrum deconvolution of αMSH-PEG-Cy5-C′ dots **(F)**, showing contributions of the absorbance of **(G)** Cy5 dye and **(H)** αMSH peptide, respectively, to the overall spectrum. αMSH, alpha melanocyte-stimulating hormone; FCS, fluorescence correlation spectroscopy; GPC, gel permeation chromatography; UV, ultraviolet.
