# Supplementary material for: A Genomic Profile of Local Immunity in the Melanoma Microenvironment Following Treatment with α Particle-Emitting Ultrasmall Silica Nanoparticles
Source: Cancer Biother Radiopharm. 2020 Aug 13;35(6):459–73. doi: 10.1089/cbr.2019.3150 (PMC7462037; doi:10.1089/cbr.2019.3150)
Supplement: Supplemental data [file Supp_Fig2.pdf]

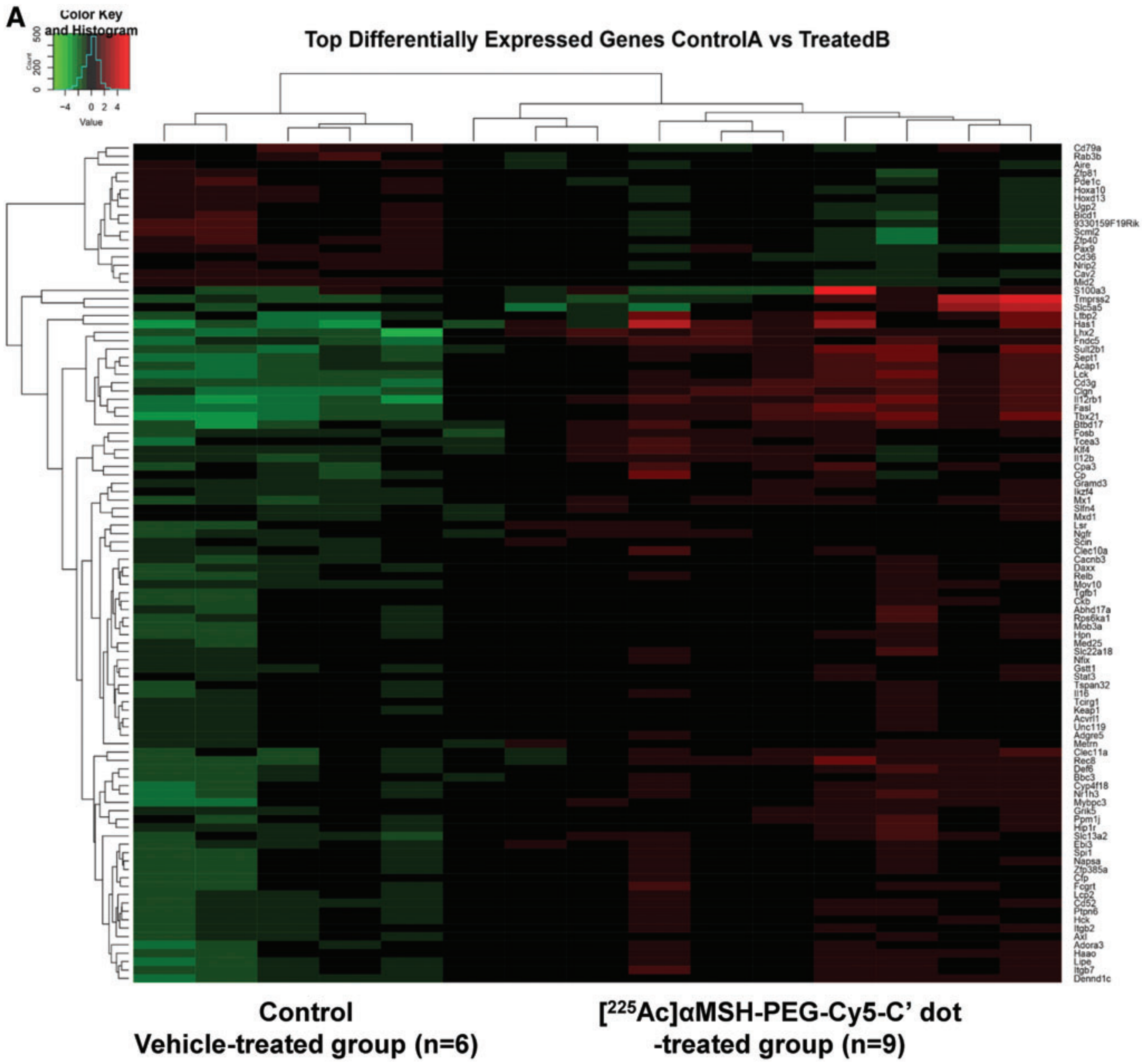

SUPPLEMENTARY FIG. S2. Top 100 differentially expressed genes in **(A)** a vehicle-treated control group versus the [^{225}Ac]αMSH-PEG-Cy5-C′ dot-treated group; **(B)** a vehicle-treated control group versus an unlabeled αMSH-PEG-Cy5-C′ dot-treated control group; and **(C)** the [^{225}Ac]αMSH-PEG-Cy5-C′ dot-treated group versus an unlabeled αMSH-PEG-Cy5-C′ dot-treated control group. The top 100 differentially expressed genes (cutoff FC = 2 and FDR = 0.05) are used. The data plot was the mean-centered normalized log2 expression of the top 100 differentially expressed genes.

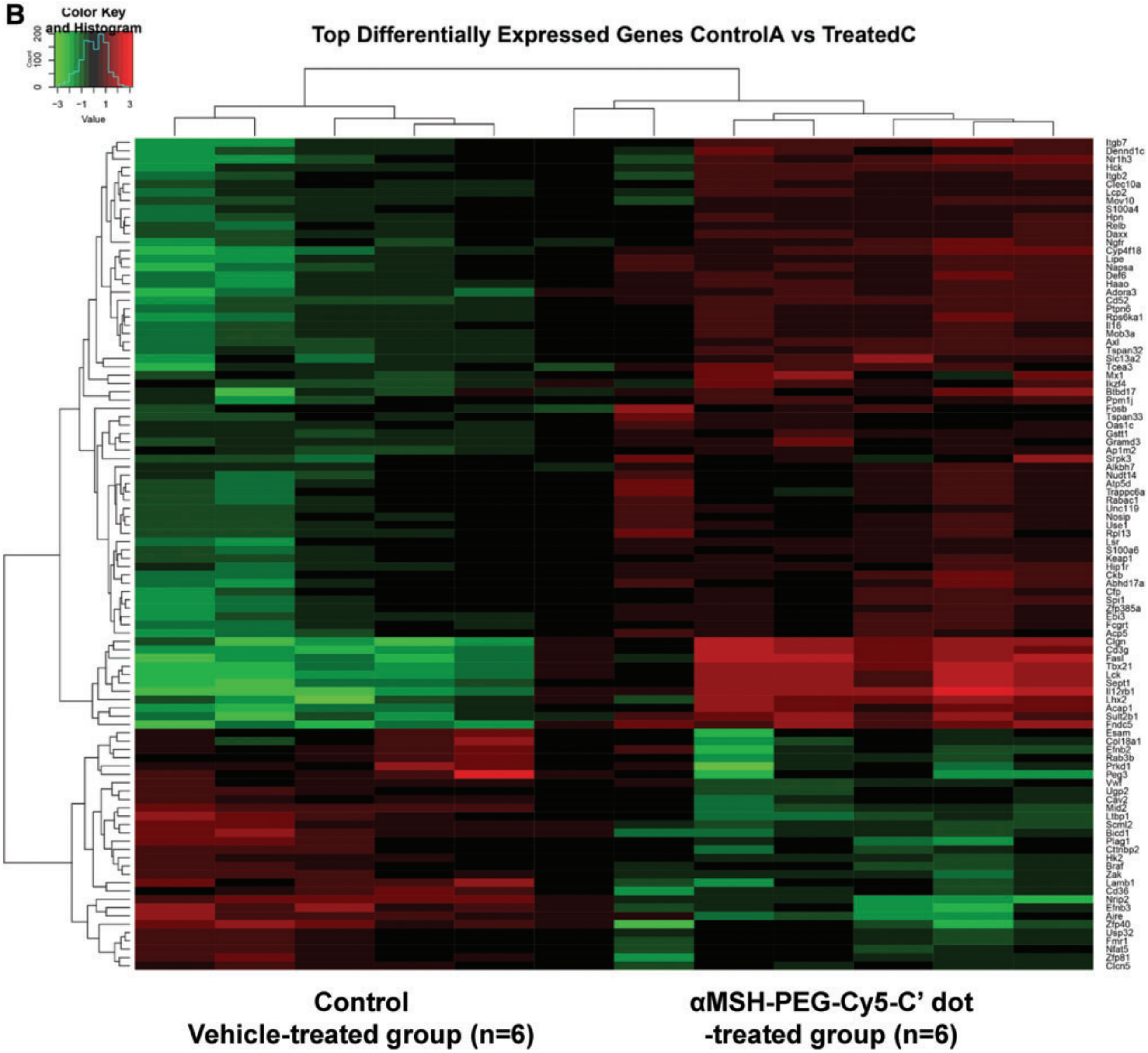

SUPPLEMENTARY FIG. S2. (Continued)

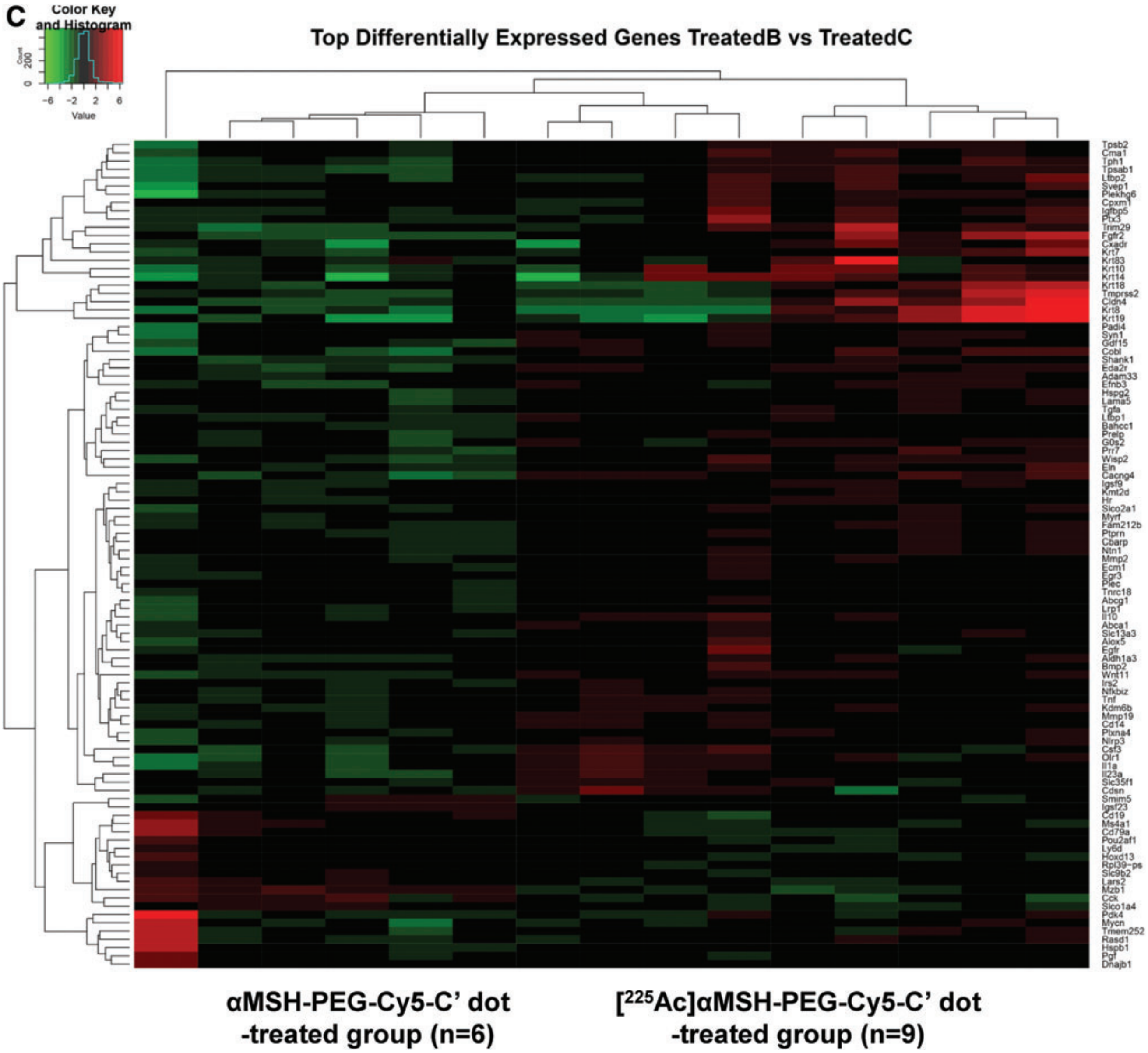

SUPPLEMENTARY FIG. S2. (Continued)
