# Supplementary material for: A Genomic Profile of Local Immunity in the Melanoma Microenvironment Following Treatment with α Particle-Emitting Ultrasmall Silica Nanoparticles
Source: Cancer Biother Radiopharm. 2020 Aug 13;35(6):459–73. doi: 10.1089/cbr.2019.3150 (PMC7462037; doi:10.1089/cbr.2019.3150)
Supplement: Supplemental data [file Supp_Fig3.pdf]

Input Sample	Mast.Cells	Neutrophil.Cells	Eosinophil.Cells	B.Cells.Memory	B.Cells.Naive	Plasma.Cells	T.Cells.CD8.Actived	T.Cells.CD8.Naive	T.Cells.CD8.Memory	M0.Macrophage	M1.Macrophage	M2.Macrophage
s_Cdot_012918_M1_B__ControlA	0	0	0	0	0.044	0.001	0	0.028	0	0.419	0.007	0.095
s_Cdot_012918_M2_B__ControlA	0	0	0	0	0.047	0.001	0.004	0.025	0.001	0.551	0.065	0.017
s_Cdot_012918_M3_B__ControlA	0	0	0	0	0.061	0	0.002	0.025	0	0.524	0.04	0.017
s_Cdot_012918_F1_B__ControlA	0.001	0	0	0	0.073	0.002	0.007	0.024	0	0.281	0.166	0.064
s_Cdot_012918_F2_B__ControlA	0	0	0	0	0.073	0.002	0.006	0.025	0	0.307	0.035	0.056
s_Cdot_012918_F3_B__ControlA	0	0	0	0	0.037	0.001	0.002	0.087	0	0.185	0.147	0.044
s_Cdot_012918_M1_D__TreatedB	0	0	0	0	0.038	0	0.017	0.037	0	0.274	0.285	0.021
s_Cdot_012918_F5_D__TreatedB	0.001	0	0	0	0.025	0	0	0.098	0.014	0	0.122	0.066
s_Cdot_012918_M2_D__TreatedB	0	0	0	0	0.046	0	0.013	0.044	0	0.194	0.278	0.033
s_Cdot_012918_M4_D__TreatedB	0	0	0	0	0.025	0	0.022	0.096	0	0.094	0.256	0.013
s_Cdot_012918_M5_D__TreatedB	0.001	0	0	0	0.069	0.001	0.001	0.061	0	0	0.242	0.101
s_Cdot_012918_F1_D__TreatedB	0.001	0	0	0	0.017	0	0.018	0.072	0	0.207	0.216	0
s_Cdot_012918_F2_D__TreatedB	0.002	0	0	0	0.036	0	0.019	0.108	0	0.102	0.267	0
s_Cdot_012918_F3_D__TreatedB	0.002	0	0	0	0.009	0	0.018	0.127	0	0.058	0.248	0.011
s_Cdot_012918_F4_D__TreatedB	0.001	0	0	0	0.016	0	0.007	0.103	0	0.064	0.243	0.007
s_Cdot_4_12_18_M2_CTL__TreatedC	0	0	0	0	0.043	0.003	0.014	0.071	0	0.006	0.156	0.081
s_Cdot_4_12_18_F3_CTL__TreatedC	0	0	0	0	0.044	0.001	0.03	0.101	0	0.01	0.22	0.036
s_Cdot_4_12_18_F2_CTL__TreatedC	0	0	0	0	0.039	0.007	0.005	0.107	0	0.103	0.181	0
s_Cdot_4_12_18_F1_CTL__TreatedC	0	0	0	0	0.025	0.001	0.016	0.143	0	0.042	0.183	0.041
s_Cdot_4_12_18_M1_CTL__TreatedC	0	0	0	0	0.142	0.006	0.008	0.072	0	0.093	0.108	0.08
s_Cdot_4_12_18_M3_CTL__TreatedC	0	0	0	0	0.037	0.01	0.009	0.076	0	0	0.134	0.162

Input Sample	Treg.Cells	T.Cells.CD4.Memory	T.Cells.CD4.Naive	T.Cells.CD4.Follicular	Th1.Cells	Th17.Cells	Th2.Cells	Monocyte	GammaDelta.T.Cells	NK.Resting	NK.Actived	DC.Actived	DC.Immature	P-value	Pearson Correlation	RMSE
s_Cdot_012918_M1_B__ControlA	0.016	0.021	0	0.01	0.006	0.001	0.007	0.11	0	0.001	0.002	0.218	0.014	0.000	0.448	0.893
s_Cdot_012918_M2_B__ControlA	0.05	0.012	0	0.006	0	0	0.002	0.105	0.003	0	0.008	0.05	0.053	0.000	0.450	0.896
s_Cdot_012918_M3_B__ControlA	0.033	0.006	0	0	0	0.006	0.004	0.105	0	0	0.014	0.08	0.081	0.000	0.479	0.883
s_Cdot_012918_F1_B__ControlA	0	0.037	0	0	0	0.051	0.002	0.146	0	0	0.018	0.128	0	0.000	0.437	0.899
s_Cdot_012918_F2_B__ControlA	0	0.055	0	0	0.003	0.086	0.011	0.107	0	0	0.015	0.218	0	0.000	0.454	0.891
s_Cdot_012918_F3_B__ControlA	0.073	0.036	0	0.027	0.023	0	0.04	0.165	0	0	0.054	0.029	0.05	0.000	0.472	0.888
s_Cdot_012918_M1_D__TreatedB	0.064	0.012	0	0.004	0.002	0	0.009	0.139	0.001	0	0.024	0.047	0.026	0.000	0.473	0.880
s_Cdot_012918_F5_D__TreatedB	0.156	0	0	0.001	0.107	0	0.012	0.111	0.001	0	0.256	0.01	0.02	0.000	0.443	0.896
s_Cdot_012918_M2_D__TreatedB	0.061	0.019	0	0.003	0.029	0	0.009	0.124	0	0	0.034	0.078	0.035	0.000	0.443	0.897
s_Cdot_012918_M4_D__TreatedB	0.067	0.017	0	0.016	0.011	0	0.031	0.233	0.002	0	0.08	0.036	0	0.000	0.467	0.884
s_Cdot_012918_M5_D__TreatedB	0.046	0.008	0	0.014	0.026	0	0.015	0.265	0	0	0.133	0.009	0.007	0.000	0.482	0.877
s_Cdot_012918_F1_D__TreatedB	0.112	0.021	0	0.004	0.004	0	0.033	0.151	0.001	0	0.117	0	0.028	0.000	0.499	0.873
s_Cdot_012918_F2_D__TreatedB	0.081	0.035	0	0.015	0.021	0	0.048	0.148	0.002	0	0.1	0.002	0.013	0.000	0.460	0.888
s_Cdot_012918_F3_D__TreatedB	0.102	0.028	0	0.039	0.02	0	0.048	0.123	0	0	0.14	0	0.027	0.000	0.461	0.887
s_Cdot_012918_F4_D__TreatedB	0.106	0.017	0	0.03	0.057	0	0.044	0.139	0.004	0	0.14	0.002	0.02	0.000	0.465	0.885
s_Cdot_4_12_18_M2_CTL__TreatedC	0.136	0.032	0	0	0.013	0.02	0.004	0.207	0	0	0.18	0	0.035	0.000	0.489	0.880
s_Cdot_4_12_18_F3_CTL__TreatedC	0.13	0.052	0	0.017	0	0	0.023	0.167	0.003	0	0.105	0	0.062	0.000	0.474	0.883
s_Cdot_4_12_18_F2_CTL__TreatedC	0.142	0.031	0	0.022	0.025	0	0.019	0.186	0.002	0	0.101	0	0.03	0.000	0.488	0.879
s_Cdot_4_12_18_F1_CTL__TreatedC	0.1	0.047	0	0.034	0.021	0	0.034	0.169	0	0	0.08	0	0.065	0.000	0.472	0.885
s_Cdot_4_12_18_M1_CTL__TreatedC	0.104	0.001	0	0.04	0.013	0.011	0.014	0.092	0	0	0.133	0.045	0.037	0.000	0.324	0.946
s_Cdot_4_12_18_M3_CTL__TreatedC	0.093	0.009	0	0.017	0.016	0	0.024	0.224	0	0	0.12	0.001	0.066	0.000	0.476	0.889

SUPPLEMENTARY FIG. S3. Tabular RNA seq data obtained from the CIBERSORT and ImmuneCC analysis of 25 different murine immune cell signatures in the 21 individual tumors that were harvested and analyzed.
