# Supplementary material for: A Genomic Profile of Local Immunity in the Melanoma Microenvironment Following Treatment with α Particle-Emitting Ultrasmall Silica Nanoparticles
Source: Cancer Biother Radiopharm. 2020 Aug 13;35(6):459–73. doi: 10.1089/cbr.2019.3150 (PMC7462037; doi:10.1089/cbr.2019.3150)
Supplement: Supplemental data [file Supp_Fig4.pdf]

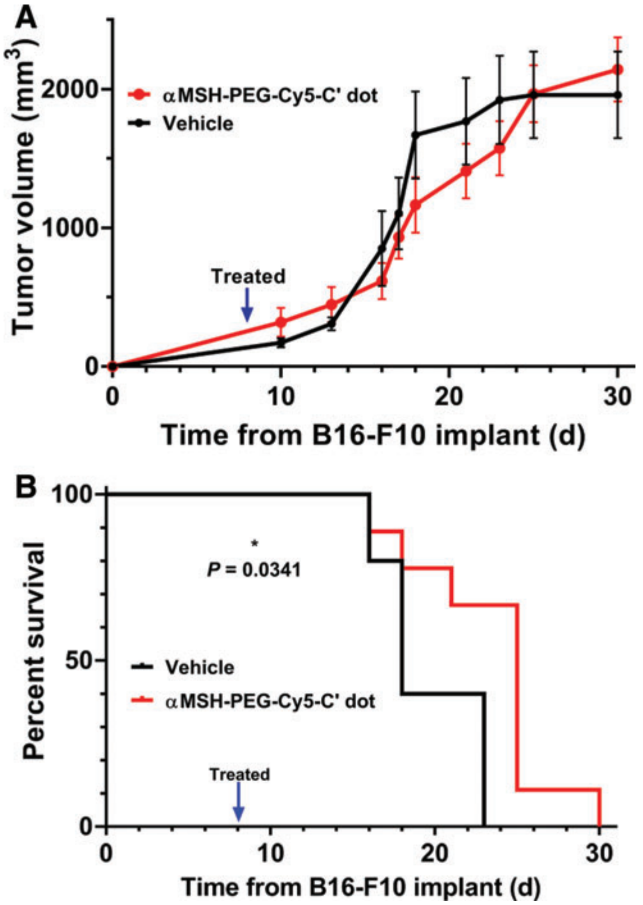

SUPPLEMENTARY FIG. S4. **(A)** Tumor volume measurements and **(B)** survival plot of B16-F10 tumor-bearing C57BL/6J mice after a single intravenous dose of 55 pmol of unlabeled (''cold'') αMSH-PEG-Cy5-C′ dot ($n = 10$) or the 1% HSA injection vehicle ($n = 5$). Data are reported as the mean ± SEM. HSA, human serum albumin; SEM, standard error of the mean.
