# Supplementary material for: A Genomic Profile of Local Immunity in the Melanoma Microenvironment Following Treatment with α Particle-Emitting Ultrasmall Silica Nanoparticles
Source: Cancer Biother Radiopharm. 2020 Aug 13;35(6):459–73. doi: 10.1089/cbr.2019.3150 (PMC7462037; doi:10.1089/cbr.2019.3150)
Supplement: Supplemental data [file Supp_Fig5.pdf]

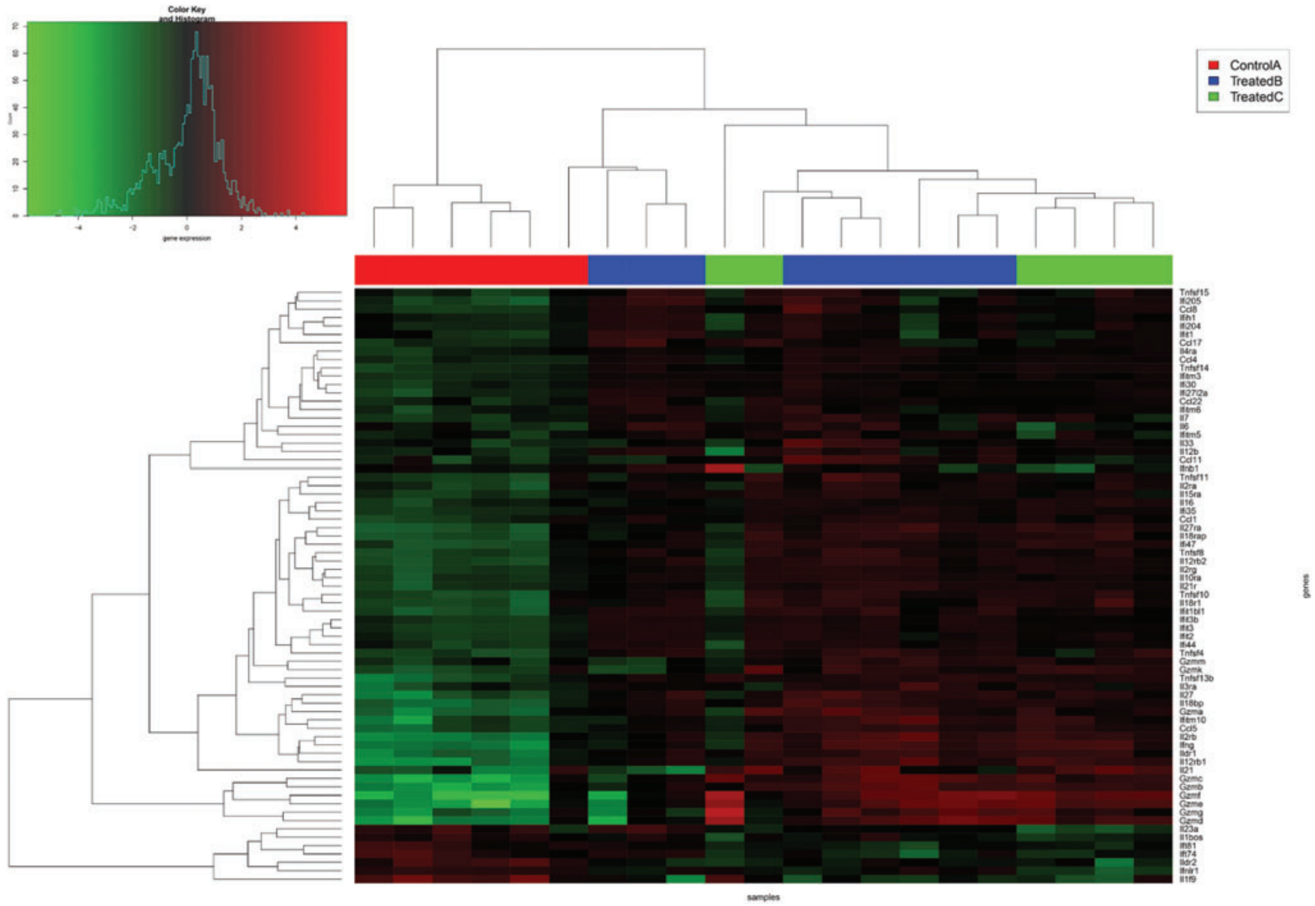

SUPPLEMENTARY FIG. S5. Heat map with cytokines that are differentially expressed in either [^{225}Ac]αMSH-PEG-Cy5-C′ dot-treated Group B versus vehicle-treated control Group A and αMSH-PEG-Cy5-C′ dot-treated Group C versus control Group A.
